# Anti-cancer pro-inflammatory effects of an IgE antibody targeting the melanoma-associated antigen chondroitin sulfate proteoglycan 4

**DOI:** 10.1038/s41467-023-37811-3

**Published:** 2023-04-25

**Authors:** Jitesh Chauhan, Melanie Grandits, Lais C. G. F. Palhares, Silvia Mele, Mano Nakamura, Jacobo López-Abente, Silvia Crescioli, Roman Laddach, Pablo Romero-Clavijo, Anthony Cheung, Chara Stavraka, Alicia M. Chenoweth, Heng Sheng Sow, Giulia Chiaruttini, Amy E. Gilbert, Tihomir Dodev, Alexander Koers, Giulia Pellizzari, Kristina M. Ilieva, Francis Man, Niwa Ali, Carl Hobbs, Sara Lombardi, Daniël A. Lionarons, Hannah J. Gould, Andrew J. Beavil, Jenny L. C. Geh, Alastair D. MacKenzie Ross, Ciaran Healy, Eduardo Calonje, Julian Downward, Frank O. Nestle, Sophia Tsoka, Debra H. Josephs, Philip J. Blower, Panagiotis Karagiannis, Katie E. Lacy, James Spicer, Sophia N. Karagiannis, Heather J. Bax

**Affiliations:** 1 St. John’s Institute of Dermatology, School of Basic & Medical Biosciences, King’s College London, London, SE1 9RT UK; 2 School of Cancer & Pharmaceutical Sciences, King’s College London, Guy’s Hospital, London, SE1 9RT UK; 3 Department of Informatics, Faculty of Natural, Mathematical and Engineering Sciences, King’s College London, Bush House, London, WC2B 4BG UK; 4 Oncogene Biology Laboratory, The Francis Crick Institute, 1 Midland Road, London, NW1 1AT UK; 5 Breast Cancer Now Research Unit, School of Cancer & Pharmaceutical Sciences, King’s College London, Guy’s Hospital, London, SE1 9RT UK; 6 Cancer Centre at Guy’s, Guy’s and St. Thomas’ NHS Foundation Trust, London, SE1 9RT UK; 7 Randall Centre for Cell and Molecular Biophysics, School of Basic and Medical Biosciences, King’s College London, London, SE1 9RT UK; 8 Asthma UK Centre, Allergic Mechanisms in Asthma, King’s College London, London, SE1 9RT UK; 9 School of Biomedical Engineering and Imaging Sciences, King’s College London, London, SE1 7EH UK; 10 Institute of Pharmaceutical Science, School of Cancer & Pharmaceutical Sciences, King’s College London, London, SE1 9NH UK; 11 Peter Gorer Department of Immunobiology, School of Immunology and Microbial Sciences, Faculty of Life Sciences and Medicine, King’s College London, London, SE1 9RT UK; 12 Centre for Gene Therapy and Regenerative Medicine, School of Basic and Medical Biosciences, Faculty of Life Sciences and Medicine, King’s College London, London, SE1 9RT UK; 13 Wolfson Centre for Age-Related Diseases, King’s College London, London, SE1 1UL UK; 14 Guy’s and St. Thomas’ Oncology & Haematology Clinical Trials (OHCT), Cancer Centre at Guy’s, London, SE1 9RT UK; 15 Department of Plastic Surgery, Guy’s and St. Thomas’ NHS Foundation Trust, London, SE1 7EH UK; 16 Skin Tumour Unit, St. John’s Institute of Dermatology, Guy’s Hospital, London, SE1 9RT UK; 17 Dermatopathology Department, St. John’s Institute of Dermatology, St. Thomas’ Hospital, London, SE1 7EH UK; 18 Sanofi US, Cambridge, Massachusetts, USA; 19 Department of Oncology, Haematology and Bone Marrow Transplantation, University Medical Centre Hamburg-Eppendorf, Hamburg, Germany

**Keywords:** Melanoma, Cancer immunotherapy, Melanoma, Translational immunology, Cancer therapy

## Abstract

Outcomes for half of patients with melanoma remain poor despite standard-of-care checkpoint inhibitor therapies. The prevalence of the melanoma-associated antigen chondroitin sulfate proteoglycan 4 (CSPG4) expression is ~70%, therefore effective immunotherapies directed at CSPG4 could benefit many patients. Since IgE exerts potent immune-activating functions in tissues, we engineer a monoclonal IgE antibody with human constant domains recognizing CSPG4 to target melanoma. CSPG4 IgE binds to human melanomas including metastases, mediates tumoricidal antibody-dependent cellular cytotoxicity and stimulates human IgE Fc-receptor-expressing monocytes towards pro-inflammatory phenotypes. IgE demonstrates anti-tumor activity in human melanoma xenograft models engrafted with human effector cells and is associated with enhanced macrophage infiltration, enriched monocyte and macrophage gene signatures and pro-inflammatory signaling pathways in the tumor microenvironment. IgE prolongs the survival of patient-derived xenograft-bearing mice reconstituted with autologous immune cells. No ex vivo activation of basophils in patient blood is measured in the presence of CSPG4 IgE. Our findings support a promising IgE-based immunotherapy for melanoma.

## Abstract (web summary)

IgE antibodies targeting cancer antigens can be used for immunotherapy. Here the authors present an IgE antibody targeting the melanoma-associated antigen, chondroitin sulphate proteoglycan 4, that recognises human melanoma, stimulates tumour cell cytotoxicity, and restricts tumour growth in humanised mouse models.

## Introduction

The tumor-associated antigen chondroitin sulfate proteoglycan 4 (CSPG4) is a highly glycosylated transmembrane proteoglycan^1^. Although CSPG4 expression has been reported in some normal tissues, including in the vascular system, skeletal and cardiac myoblasts, and chondroblasts^2^, CSPG4 is overexpressed in several solid tumors, including malignant melanoma, subsets of breast cancer, mesothelioma, and neuroblastoma. Therefore, CSPG4 is considered a promising target for cancer-targeting immunotherapies^1^. Several CSPG4 targeting therapeutics have been evaluated in pre-clinical and clinical studies; showing preliminary efficacy and favorable safety profiles^3–8^.

Despite substantial progress with the approval of checkpoint inhibitor antibody immunotherapies, the 5-year survival rates remain poor (<55%) for patients with late stage disease^9^. Many patients’ tumors do not respond to existing immune and targeted therapies or acquired resistance develops quickly^10^. Checkpoint inhibitor antibody immunotherapies are designed to activate immune cells irrespective of their antigen reactivity. Therefore, immunotherapies targeting a melanoma-associated antigen, such as CSPG4, may effectively direct immune cells against cancer and address a significant unmet need.

Immunotherapeutic antibodies used to treat cancer belong to the immunoglobulin G (IgG) antibody class (most often IgG1). However, tumor antigen-specific immunoglobulin E (IgE) antibodies may offer significant potential advantages and have shown favorable results in both in vitro and in vivo models^11–18^. Strong adaptive immune responses triggered by IgE, may protect from cancer growth^19–23^. IgE has a high affinity for its cognate high-affinity Fcε receptor (FcεRI) and slower dissociation compared to IgG for its respective Fcγ receptors. The high affinity of IgE for its Fcε receptors may translate to long retention on immune effector cells in tissues, such as within a tumor, for long periods (up to 14 days)^24,25^. Unlike IgG, IgE has no known inhibitory Fc receptors. Studies to-date have also demonstrated that localized immune cell activation and release of mediators can uniquely activate immune cells to perpetuate anti-tumoral activities^26^. Pre-clinical studies of the first anti-cancer IgE, MOv18 IgE (targeting the cancer antigen folate receptor alpha, FRα) have now been translated to a first-in-human, first-in-class Phase I clinical trial (NCT02546921)^27^. However, an IgE class antibody targeting a melanoma-associated antigen has not yet been evaluated.

Here we confirm the expression of CSPG4 in human melanomas compared with normal tissues and demonstrate that a recombinant anti-CSPG4 antibody (clone 225.28), generated with human IgE class constant domains, binds human CSPG4-expressing cancer cells and melanoma tissues including lymph, and distant metastases. We assess the anti-tumor effects of CSPG4 IgE in vitro and in vivo in human melanoma xenograft models, and a melanoma patient-derived xenograft (PDX) model. Cellular immunity in these animal models is reconstituted using human immune effector cells from healthy volunteers and from patients with melanoma. We study the effects of IgE Fc-mediated immune effector cell functions, activation and signaling pathways ex vivo and in vivo. Furthermore, we evaluate CSPG4 IgE in an ex vivo basophil activation assay in whole patient blood to consider the potential of type I hypersensitivity.

## Results

### CSPG4 over-expression across malignant melanoma tissues, and engineering and characterization of CSPG4 IgE

**Fig. 1:**
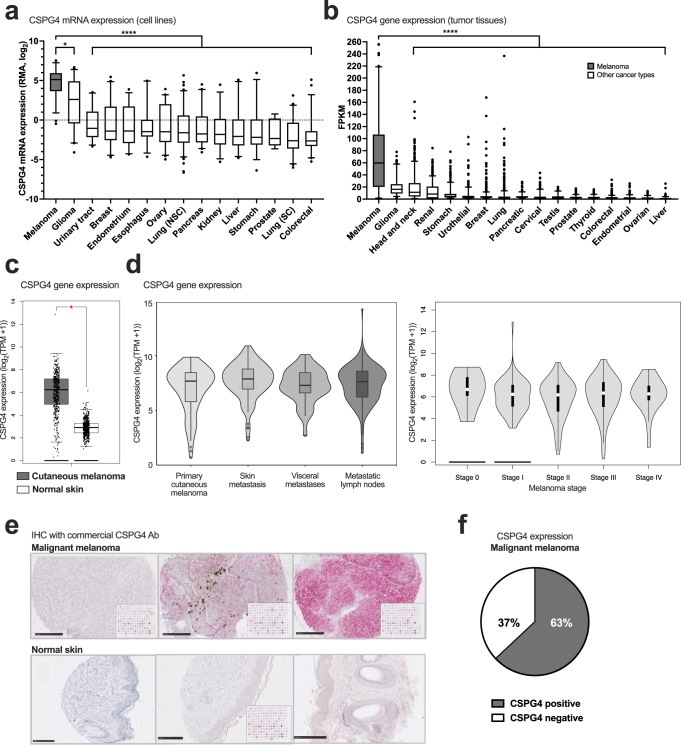
CSPG4 expression in malignant melanoma and normal tissues. **a** CSPG4 mRNA expression, derived from RNAseq data, across cell lines of different cancer cell types. *n* represents the number of cell lines (data from Cancer Cell Line Encyclopedia (CCLE), portals.broadinstitute.org/ccle, *n* = 56, *n* = 59, *n* = 26, *n* = 57, *n* = 28, *n* = 27, *n* = 48, *n* = 127, *n* = 41, *n* = 32, *n* = 26, *n* = 37, *n* = 8, *n* = 50, and *n* = 58, respectively) (*p* = 0.0156 and *p* ≤ 0.0001). **b** CSPG4 gene expression in tissues across cancer types (data and images from Human Protein Atlas, v20.proteinatlas.org^28, 29^, *n* = 153, *n* = 499, *n* = 877, *n* = 354, *n* = 406, *n* = 1075, *n* = 994, *n* = 176, *n* = 291, *n* = 134, *n* = 494, *n* = 501, *n* = 597, *n* = 541, *n* = 373, and *n* = 365 samples, respectively per cancer type; *p* ≤ 0.0001). **c** Comparison of CPSG4 gene expression between cutaneous melanoma and normal skin tissues (from GEPIA)^67^ (*n* = 461 and *n* = 558, respectively). TPM = transcripts per million. **d** CPSG4 gene expression across primary cutaneous melanoma lesions, skin metastases, visceral metastases and metastatic lymph nodes (left; TCGA-SKCM data was obtained from xenabrowser.net^68^, *n* = 103, *n* = 116, *n* = 36, *n* = 208, respectively), and across disease stages of melanoma (right; from GEPIA^67^). **e** Representative immunohistochemical (IHC) images of malignant melanoma samples showing low, intermediate, and high CSPG4 expression (pink staining, left to right) respectively, and normal skin tissue (showing no/low CSPG4 expression). Samples were stained with a commercially sourced anti-human CSPG4 antibody and CSPG4 expression was detected by alkaline phosphatase (AP; pink) staining. Nuclei were stained with hematoxylin (blue). Scale bar = 250 μm. **f** Quantitative analyses of CSPG4 expression detected in human melanoma and non-malignant tissues by IHC: expression was detected in 63% of all melanoma tissues (*n* = 428). Boxes denote 25th to 75th percentile with median line. Whiskers mark the minima 5th percentile to the maxima 95th percentile. Data shown as mean ± SEM. Source data are provided as a Source Data file. Kruskal–Wallis (**a**, **b**, **d** left), One-way ANOVA (**d** right), Student’s *t* test (**c**): *p ≤ 0.05; ****p ≤ 0.0001.

Transcriptomic analyses of publicly available datasets confirmed significantly higher levels of CSPG4 gene expression in melanomas compared with other tumor types among several tumor cell lines (*n* = 8–127 per cancer type) and human cancer tissues (*n* = 102–1075 per cancer type)^28,29^ (Fig. 1a, b, respectively), and in cutaneous melanomas (*n* = 461) compared to normal skin (*n* = 558) tissues (Fig. 1c). Furthermore, CSPG4 gene expression was measured across primary and metastatic disease (skin, visceral and lymph node metastases) (Fig. 1d, left, *n* = 36–208), and across all stages of melanoma (Fig. 1d, right). Immunohistochemistry (IHC) evaluations using a mouse anti-human CSPG4 antibody (detected by alkaline phosphatase (AP, pink)) in human melanomas (*n* = 428, Fig. 1e and Supplementary Fig. 1), and several normal tissues (*n* = 389) indicated detectable CSPG4 protein expression in 63% of malignant melanomas (*n* = 428, Fig. 1f).

**Fig. 2:**
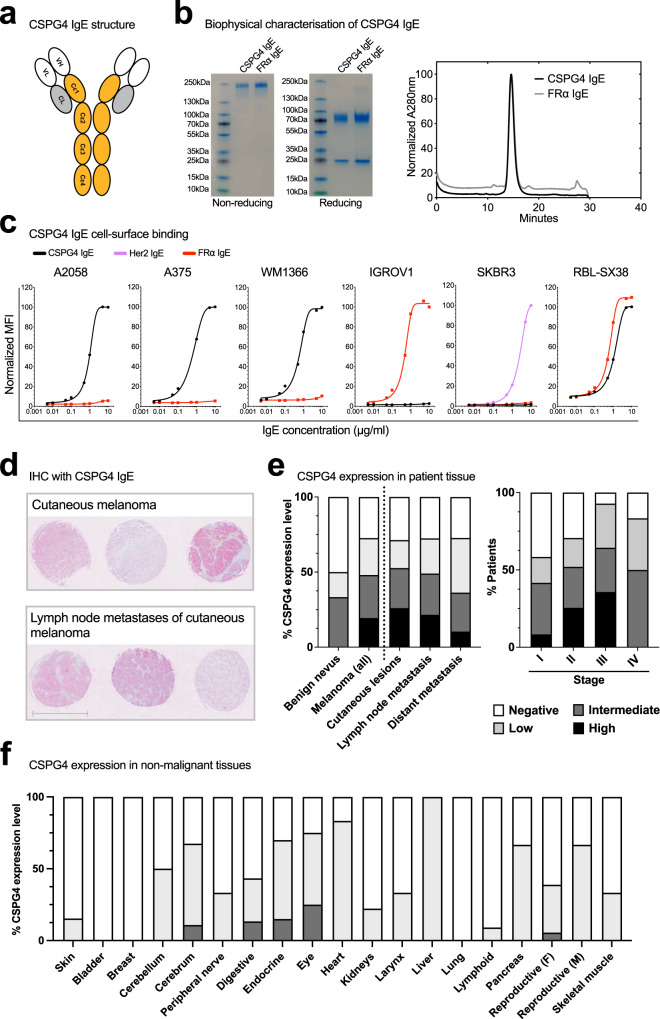
Generation, biophysical characterization, and cancer specificity of CSPG4 IgE. **a** Structure of CSPG4 IgE: CSPG4-specific variable domains (white), constant heavy chain domains (orange) and constant light chain domains (gray). **b** SDS-PAGE (non-reduced (left) and reduced (middle) conditions) and size-exclusion HPLC (right) analyses confirmed comparable size and purity of CSPG4 IgE in relation to a previously engineered anti-Folate Receptor alpha (FRα) IgE. **c** Flow cytometric analyses of CSPG4 IgE (black line) confirmed binding to CSPG4-expressing human melanoma cell lines (A2058, A375, and WM1366), but not to FRα- or Her2-expressing cancer cells (IGROV1 and SKBR3, respectively). Antibody Fc-binding to human FcεRI on RBL-SX38 rat basophilic leukemia cells was also demonstrated (representative data). **d** Representative immunohistochemical images of cutaneous melanoma and lymph node metastases specimens stained with the engineered CSPG4 IgE (detected by alkaline phosphatase (AP; pink)) staining; nuclei were stained with hematoxylin (blue). Scale bar = 1 mm. **e** Quantitation of CSPG4 expression detected by IHC using the engineered CSPG4 IgE: the clone detected CSPG4 low/intermediate expression in 50% of benign nevus samples (*n* = 18) and variable high to low expression levels in 72–73% of malignant specimens (*n* = 468; including further analysis of antibody binding to cutaneous lesions, lymph node metastases and distant metastases (*n* = 150, *n* = 75 and *n* = 77, respectively)) (right), and high to low CSPG4 expression in 58–93% of melanoma lesions across stages I–IV (left; *n* = 12, *n* = 102, *n* = 14 and *n* = 6, respectively). **f** CSPG4 IgE IHC staining indicated absent or low/intermediate expression of CSPG4 in non-malignant tissue specimens (*n* = 297). Source data are provided as a Source Data file.

The anti-tumor functions of the first-in-class MOv18 IgE have been previously reported, and point to the involvement of IgE in engaging and reprogramming Fcε receptor-expressing immune effector cells, such as monocytes and macrophages, in the tumor microenvironment (TME)^11–13,15,30,31^. We generated a monoclonal antibody with human IgE constant domains and mouse variable regions from a CSPG4-specific clone (Fig. 2a)^32^. Affinity-purified CSPG4 IgE showed comparable biophysical properties to those of MOv18 (FRα IgE)^33^. SDS polyacrylamide gel electrophoresis (SDS-PAGE), under reduced and non-reduced conditions, and size-exclusion high performance liquid chromatography (SEC-HPLC) confirmed a monodisperse product with subunit composition and molecular mass consistent with that of equivalent IgEs^33^, and purity typically above 95% (Fig. 2b).

Flow cytometric and IHC analyses (Fig. 2c–f) demonstrated dose-dependent binding of CSPG4 IgE to human melanoma cell lines known to express human CSPG4 (A2058, A375, WM1366)^5,34^. As expected, we did not detect binding to IGROV1 ovarian or SKBR3 breast cancer cells (neither express CSPG4, but overexpress tumor antigens FRα and human epidermal growth factor receptor 2 (Her2), respectively, confirmed by binding of target antigen-specific IgEs) (Fig. 2c). Furthermore, comparably to a commercial anti-CSPG4 antibody, CSPG4 IgE binding was absent in three A375 and three A2058 CSPG4-knock-out cell lines (Supplementary Fig. 2). Sequence similarity between the human and mouse CSPG4 antigen amino acid sequences is 83.55% (Supplementary Fig. 3), however CSPG4 IgE did not bind to mouse CSPG4-expressing tumor-derived mouse melanoma cell lines (Supplementary Fig. 4, mouse CSPG4 expression confirmed with an anti-mouse CSPG4 antibody by flow cytometric and Western Blot evaluations). Comparably to FRα IgE, CSPG4 IgE bound to human FcεRI-expressing rat basophilic leukemia cells (RBL-SX38) (Fig. 2c, right). In concordance with IHC evaluations using a commercial mouse antibody clone, CSPG4 IgE showed positive staining of ~70–75% of all malignant melanomas (*n* = 468), and when divided into cutaneous lesions, lymph node and distant metastases (*n* = 302). Benign nevi (*n* = 18) showed low/intermediate CSPG4 expression detected with CSPG4 IgE (Fig. 2d, e, left), and CSPG4 expression was retained across all stages of melanoma (Fig. 2e, right). Binding to normal human tissues (*n* = 297) was either negative or low, other than the cerebrum, digestive, endocrine, eye, and female reproductive tissues where low or intermediate CSPG4 expression was observed in a proportion of tissues (Fig. 2f).

Thus, CSGP4 expression was detected in malignant melanomas and showed low and restricted distribution in normal tissues. The chimeric CSPG4 IgE and a mouse anti-human CSPG4 clone showed comparable binding to human tissues by IHC, and recognized CSPG4-expressing melanoma cells.

### CSPG4 IgE can exert in vitro anti-tumoral functions

**Fig. 3:**
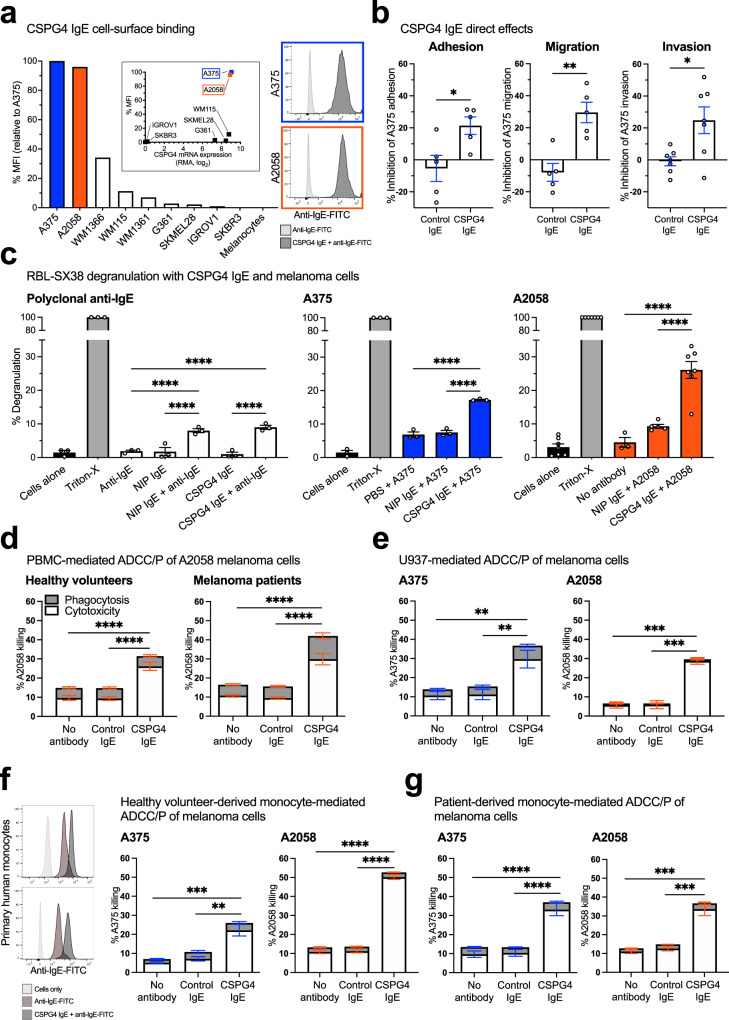
Anti-tumor and Fc-mediated effector functions of CSPG4 IgE in vitro. **a** Left: CSPG4 expression levels by human cancer cell lines and melanocytes (as control cells) normalized relative to the mean fluorescent intensity (MFI) of CSPG4 IgE binding to A375 melanoma cells. Inset: Analyses of CSPG4 mRNA expression data in cell lines extracted from the CCLE database (Cancer Cell Line Encyclopedia (CCLE), portals.broadinstitute.org/ccle) and cell-surface binding of CSPG4 IgE (*r* = 0.2680). Right: Representative histograms for the highest expressing A375 (blue) and A2058 (orange) cells (light gray, anti-IgE-FITC only; dark gray, CSPG4 IgE + anti-IgE-FITC). **b** Treatment of A375 melanoma cells with CSPG4 IgE resulted in moderate restrictions in cancer cell adhesion (*n* = 5), migration (*n* = 5) and invasion (*n* = 7) compared with an isotype IgE control (Control IgE) (*p* = 0.0263, *p* = 0.0022, and *p* = 0.0131, respectively). **c** CSPG4 IgE-mediated degranulation of FcεRI-expressing RBL-SX38 cells when cross-linked by polyclonal anti-IgE (left, *n* = 3) or with CSPG4-expressing cancer cells (A375, middle, *n* = 3; A2058, right, *n* = 5). **d**–**g** Compared to cells alone and treatment with isotype control IgE, CSPG4 IgE-mediated significant levels of antibody-dependent cell-mediated cytotoxicity (ADCC; white bars; ADCP; gray bars) of CSPG4-expressing melanoma cell lines (A2058, orange; A375, blue). **d** ADCC/ADCP by healthy volunteer and melanoma patient-derived PBMCs (healthy volunteer: left, *n* = 17, *p* ≤ 0.0001; melanoma patients: right, *n* = 14, *p* ≤ 0.0001). **e** ADCC/ADCP by U937 monocytic cells (A375: left, *n* = 10, *p* = 0.0075 and *p* = 0.0038; A2058: right, *n* = 10, *p* = 0.0008 and *p* = 0.0004). **f** Left: Flow cytometric histograms show cell-surface detection of endogenous bound IgE (anti-IgE-FITC) and of CSPG4 IgE (CSPG4 IgE + anti-IgE-FITC) to primary human monocytes from two healthy volunteers; Right: ADCC/ADCP by healthy volunteer monocytes (A375: left, *n* = 8, *p* = 0.0004 and *p* = 0.0089; A2058: right, *n* = 4, *p* ≤ 0.0001). **g** ADCC/ADCP by patient-derived monocytes (A375: left, *n* = 9, *p* ≤ 0.0001; A2058: right, *n* = 3, *p* = 0.0010 and *p* = 0.0010). No phagocytosis (ADCP, gray bars) was triggered by CSPG4 IgE above controls. Data shown as mean ± SEM. Source data are provided as a Source Data file. Two-tailed Student’s *t* test (**b**), One-way ANOVA (**c**–**e** right, **f**, **g**), Kruskal–Wallis test (**e** left): *p ≤ 0.05; **p ≤ 0.01; ***p ≤ 0.001; ****p ≤ 0.0001.

We next evaluated the potential anti-tumoral functions of CSPG4 IgE. We analyzed the relative expression of CSPG4 in human melanoma cell lines (A375, A2058, WM1366, WM115, WM1361, G361, and SKMEL28) in comparison to cells not known to express CSPG4 (IGROV1 ovarian and SKBR3 breast cancer cells, and primary non-malignant melanocytes) (Fig. 3a). We selected the high CSPG4-expressing A375 and A2058 cells (human CSPG4 expression confirmed with commercial antibody, Supplementary Fig. 2) in subsequent functional analyses.

It was previously reported that high expression of CSPG4 and its downstream signaling pathways in melanoma cells may contribute to tumor progression^1,35^. Since the Fab-mediated and Fc-mediated effector functions of anti-tumor IgE antibodies directed against tumor cells may restrict cancer cell growth^11–13,15–18,24,36–38^, we investigated whether CSPG4 IgE could impair cancer cell function in vitro. Firstly, we studied the Fab-mediated direct effects of our antibody on cancer cell functions, in the absence of cross-linking or effector cells. A375 cell adhesion, migration, and invasion were partly inhibited by CSPG4 IgE, compared a non-specific isotype control (Fig. 3b). Furthermore, CSPG4 IgE bound by its Fc domains to RBL-SX38 cells and cross-linked by polyclonal anti-IgE could trigger cell degranulation (measured by β-hexosaminidase release), similarly to positive control cross-linked hapten-specific (NIP) IgE. CSPG4 IgE triggered significantly greater degranulation than the non-specific isotype control, NIP IgE, in the presence of high CSPG4-expressing A375 and A2058 melanoma cells (Fig. 3c). While CSPG4 IgE triggered significant degranulation in the presence of WM1366 cells expressing intermediate levels of CSPG4, the antibody did not trigger degranulation in the presence of low target-expressing WM1361 cells or non-CSPG4-expressing SKBR3 breast cancer cells (Supplementary Fig. 5). As expected, control Her2 IgE triggered significant degranulation in the presence of SKBR3 cells which express high levels of the Her2 target of Her2 IgE (Supplementary Fig. 5).

Furthermore, we investigated whether CSPG4 IgE could exert Fc-mediated effector killing of cancer cells^39^ (Fig. 3d–g, Supplementary Fig. 6a). CSPG4 IgE triggered significant levels of antibody-dependent cellular cytotoxicity (ADCC) of A2058 and A375 melanoma cells above isotype control IgE by healthy volunteer and melanoma patient-derived peripheral blood mononuclear cells (PBMCs) (Fig. 3d, left and right, respectively) and human monocytic U937 cells (Fig. 3e). Binding of CSPG4 to unoccupied Fcɛ receptor I (FcɛRI) on primary human monocytes was confirmed by flow cytometry (Fig. 3f, left). CSPG4 IgE-mediated ADCC of A375 and A2058 melanoma cells was triggered by monocytes derived from both healthy participants (Fig. 3f, right) and patients with melanoma (Fig. 3g). Contrastingly, CSPG4 IgE-engaged effector cells did not trigger ADCC of intermediate CSPG4-expressing WM1366 melanoma cells, or of non-expressing primary human melanocytes (Supplementary Fig. 6b, c, respectively). Together these findings suggest that ADCC functions are dependent on the antigen expression by target cells. A function for monocytes as effector cells was further supported by ADCC mediated by CSPG4 IgE in the presence of PBMCs, but not in the presence of monocyte-depleted PBMCs (Supplementary Fig. 6d, e). Additionally, the need for antibody Fc-mediated activation of effector cells was supported by the inhibition of ADCC by a PTK2 inhibitor which blocks downstream signaling on human monocytic cells (Supplementary Fig. 6f).

CSGP4 IgE exerted direct effects against melanoma cells compared to non-specific IgE and engaged human healthy volunteer and melanoma patient-derived effector cells to trigger in vitro effector functions, tumor cell cytotoxicity and degranulation.

### CSPG4 IgE-mediated upregulation of immune mediators in human monocyte supernatants and significantly increased expression of pro-inflammatory cell-surface markers in monocytes

**Fig. 4:**
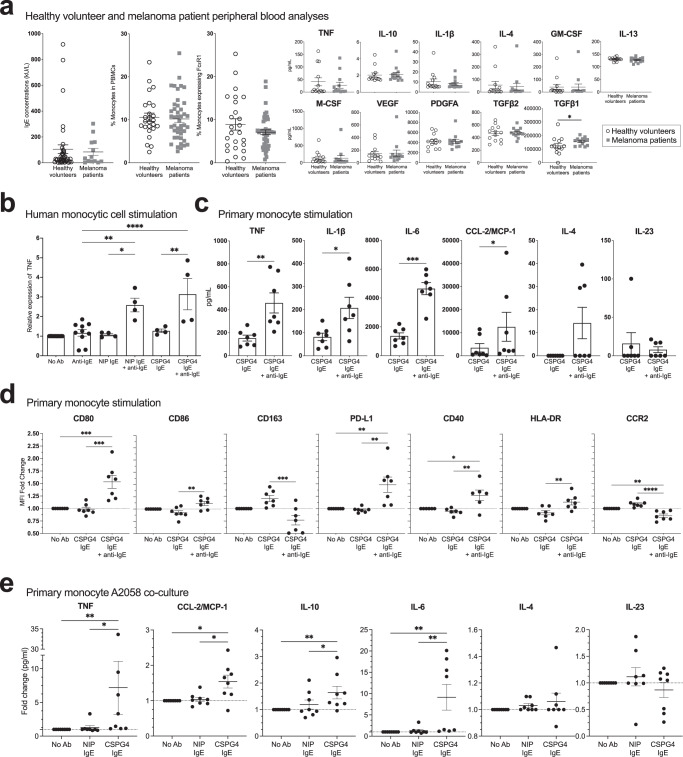
Cross-linking of CSPG4 IgE on the surface of human monocytes promotes secretion of pro-inflammatory cytokines and enhanced expression of co-stimulatory cell-surface markers. **a** Serum analysis of total IgE levels in healthy volunteers (*n* = 38) and melanoma patients (*n* = 13). Flow cytometric analysis of % monocytes in total PMBCs (healthy volunteers, *n* = 25; melanoma patients, *n* = 44) and % of monocytes expressing FcɛRI (healthy volunteers, *n* = 25; melanoma patients, *n* = 46). Concentrations of cytokines and chemokines measured in the sera of sex-matched healthy volunteers (*n* = 13) and melanoma patients (*n* = 13) (*p* = 0.0387). **b** Quantitative PCR (qPCR) analysis of TNF expression following cross-linking of human IgE bound to human monocytic U937 cells (*n* = 4 independent experimental repeats). Anti-IgE vs. CSPG4 IgE + anti-IgE, *p*≤0.0001; Anti-IgE vs. NIP IgE + anti-IgE, *p* = 0.0039; NIP IgE vs. NIP IgE + anti-IgE, *p* = 0.0113; CSPG4 IgE vs. CSPG4 IgE + anti-IgE, *p* = 0.0014. **c** Cytokine and chemokine secretion in primary monocyte culture supernatants following cross-linking of IgE (*n* = 7 healthy volunteers; *p* = 0.0085, *p* = 0.0152, *p* = 0.0001, and *p* = 0.0156, respectively). **d** Flow cytometric analysis (MFI change) in the expression levels of cell-surface markers of healthy volunteer monocytes untreated or stimulated with CSPG4 IgE with and without cross-linking with anti-IgE (*n* = 7 healthy volunteers; CD80: *p* = 0.0004 and *p* = 0.0003, CD86: *p* = 0.0038, CD163: *p* = 0.0005, PD-L1: *p* = 0.0033 and *p* = 0.0021, CD40: *p* = 0.0227 and *p* = 0.0060, HLA-DR: *p* = 0.0046, CCR2: *p*-0.0015 and ≤0.0001). **e** Cytokine and chemokine secretion in supernatants from primary human monocyte and A2058 cancer cell co-cultures in the presence of CSPG4 IgE or control NIP IgE (*n* = 8 healthy volunteers; TNF: *p* = 0.0053 and *p* = 0.0121, CCL-2/MCP-1: *p* = 0.0103 and *p* = 0.0110, IL-10: *p* = 0.0093 and *p* = 0.0492, IL-6: *p* = 0.0081 and *p* = 0.0081). Data shown as mean ± SEM. Source data are provided as a Source Data file. Mann–Whitney (**a** left), Two-tailed Student’s *t* test (**a** right, **c**), One-way ANOVA (**b**, **d**, **e**): *p ≤ 0.05; **p ≤ 0.01; ***p ≤ 0.001; ****p ≤ 0.0001.

Previous studies suggested that IgE stimulation of immune effector cells such as ovarian cancer patient monocytes may potentiate pro-inflammatory signals^31^. Here we aimed to evaluate these functions in the context of CSPG4 IgE and melanoma. In healthy volunteer and melanoma patient blood we investigated; IgE titers (*n* = 38 and *n* = 13, respectively) and the proportion of monocytes overall within PBMCs (*n* = 25 and *n* = 44, respectively) and of monocytes expressing the high-affinity IgE Fc receptor FcɛRI (*n* = 25 and *n* = 46, respectively). We found no significant differences in any of these parameters (Fig. 4a, left three graphs). Similarly, when evaluating chemokine and cytokine profiles in the sera of healthy volunteers and melanoma patients, no significant differences were measured in TNF, IL-10, IL-1β, IL-4, GM-CSF, IL-13, M-CSF, VEGF, PDGFA, and TGFβ2, while TGFβ1 levels were significantly higher in the melanoma cohort (Fig. 4a, right graphs).

Cross-linking of FcɛRI-bound CSPG4 IgE with a polyclonal anti-IgE to mimic immune complex formation on the surface of human monocytic U937 cells resulted in the upregulation of TNF (Fig. 4b), in concordance with previous findings^13^. Subsequently, in supernatants from human monocytes stimulated with CSPG4 IgE (Fig. 4c), we detected significant increases in secreted TNF, IL-1β, IL-6, and CCL-2/MCP-1 when CSPG4 IgE was cross-linked compared with cells given IgE alone, while no significant differences were observed in IL-4 and IL-23 titers. Flow cytometric analyses of monocyte cell-surface markers (Fig. 4d) showed that IgE cross-linking resulted in significantly increased expression of the co-stimulatory and activation molecules CD80, CD86, PD-L1, CD40, and HLA-DR, and decreased expression of the scavenger receptor CD163 and of CCR2, compared with cells given IgE alone. Cross-linking of CSPG4 IgE bound to primary monocytes in an antigen-specific manner, in co-cultures with CSPG4-expressing A2058 cancer cells also triggered significantly increased secretion of TNF, CCL-2/MCP-1, IL-10, and IL-6, but not of IL-4 and IL-23, compared with non-specific IgE control co-cultures (Fig. 4e).

Cross-linking of CSPG4 IgE on the surface of human monocytes significantly increased the production and expression of pro-inflammatory cytokines and co-stimulatory cell-surface markers.

### CSPG4 IgE restricted melanoma tumor growth in vivo

**Fig. 5:**
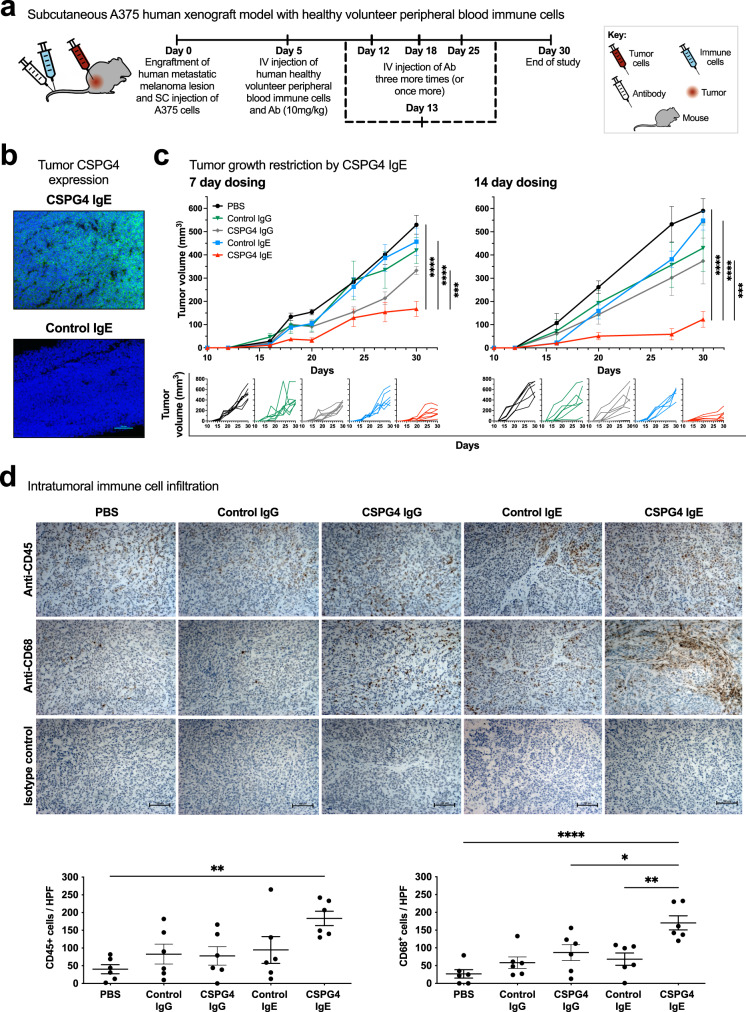
CSPG4 IgE treatment can restrict tumor growth and induce human immune cell infiltration in a subcutaneous A375 in vivo model engrafted with healthy volunteer immune cells. **a** Design and dosing regimen for in vivo model. SC = subcutaneous, IV = intravenous, Ab = antibody. **b** Immunofluorescence images of CSPG4 expression (green) in established A375 melanoma xenografts grown subcutaneously in immunocompromised mice. Frozen tumor sections were labeled with CSPG4 IgE (top, green) or isotype control IgE antibody (bottom), followed by fluorescently conjugated anti-human IgE. DAPI (blue): nuclear staining; 10× magnification; scale bar = 150 μm. **c** CSPG4 IgE significantly inhibited the growth of subcutaneous A375 tumors in immunodeficient mice engrafted with human peripheral blood immune cells. Mice challenged with subcutaneous melanomas were treated every 7 days (left) or 14 days (right) with either vehicle alone (PBS, black), CSPG4 IgE (red), isotype control IgE (blue), CSPG4 IgG (gray), isotype control IgG (green) (7 day dosing: *n* = 7 mice per group; 14 day dosing: PBS, CSPG4 IgE and CSPG4 IgG; *n* = 7, MOv18 IgE and MOv18 IgG; *n* = 6 mice per group). Inset graphs below show tumor growth curves for individual animals. Full statistical analyses shown in Supplementary Tables 1, 2. **d** Compared to CSPG4 IgG and control-treated mice, immunohistochemical studies showed elevated levels of human CD45^+^ leukocytes and CD68^+^ macrophages observed in subcutaneous A375 tumors excised from animals treated with CSPG4 IgE; top: representative images, ×10 magnification, scale bar = 100 μm; bottom: number of positive cell infiltrates per animal (*n* = 6; CD45^+^: *p* = 0.0072, CD68^+^: *p* ≤ 0.0001, *p* = 0.0201, *p* = 0.0036) (each value was derived from 3 independent images per high-power field (HPF)). Data shown as mean ± SEM. Source data are provided as a Source Data file. Two-way ANOVA (**c**), One-way ANOVA (**d**): *p ≤ 0.05; **p ≤ 0.01; ***p ≤ 0.001; ****p ≤ 0.0001.

We investigated whether CSGP4 IgE could restrict human melanoma growth in vivo in immunodeficient (NOD/scid/IL-2R γ−/−) mice. Since human IgE does not react with mouse FcεR-expressing immune cells, mice were engrafted with healthy volunteer peripheral blood immune cells and challenged subcutaneously with A375 melanoma cells (Fig. 5a). Tumors excised after 30 days retained CSPG4 expression (Fig. 5b, Supplementary Fig. 7a) and we confirmed human immune cell engraftment by detection of human CD45^+^ leukocytes in mouse spleens (Supplementary Fig. 7b). Compared to the IgE isotype control and the corresponding CSPG4 IgG treatment, CSPG4 IgE given either at 7- or 14-day intervals significantly restricted the growth of subcutaneous human melanoma xenografts in mice (Fig. 5c), despite markedly faster clearance of CSPG4 IgE from the circulation compared to CSPG4 IgG (Supplementary Fig. 7c).

Consistent with previous in vivo evaluations of anti-FRα IgE^11,13^, immunohistochemical analysis of excised tumors showed infiltration of human CD45^+^ leukocytes, and significantly higher CD68^+^ macrophage infiltration in tumors from mice treated with CSPG4 IgE, compared to tumors from mice given isotype control IgE or CSPG4 IgG (Fig. 5d). Furthermore, human tumor growth restriction by CSPG4 IgE in human PBMC engrafted mice was ablated by depletion of monocytes from PBMCs prior to injection (Supplementary Fig. 7d).

**Fig. 6:**
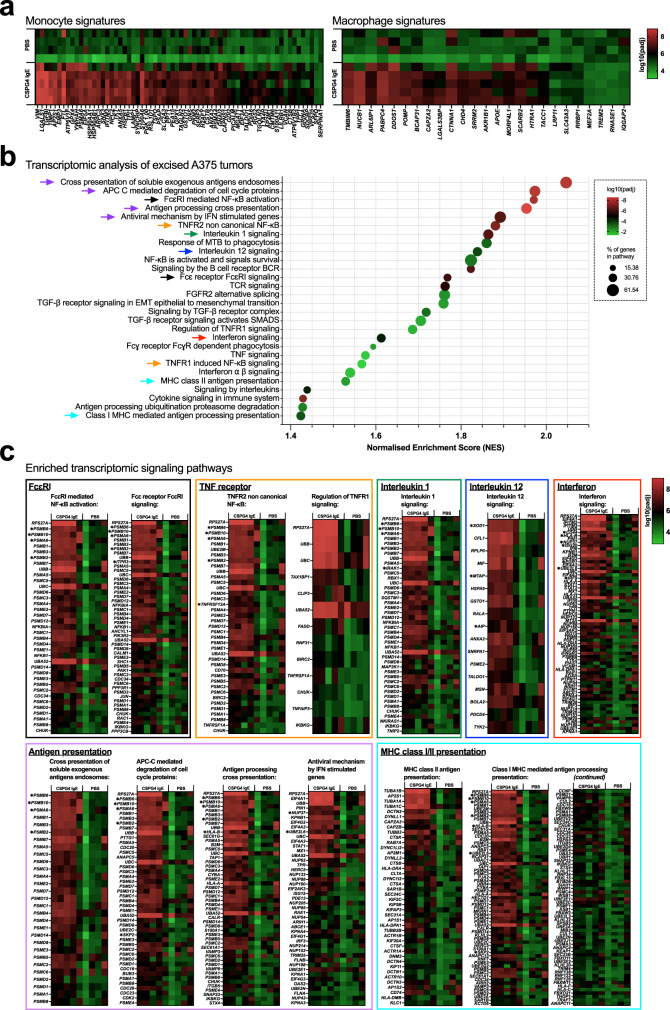
Monocyte and macrophage signatures, and activation of Fcɛ receptor and pro-inflammatory immune pathways, with CSPG4 IgE treatment in vivo. Gene expression and enriched pathways were studied in A375 subcutaneous tumors from mice treated intravenously with CSPG4 IgE (*n* = 4) or PBS (*n* = 5). **a** Significantly differential expression of monocyte and macrophage gene signatures (signatures annotated as per Li et al.^73^). **b** Differentially expressed genes were identified using the package limma, ranked according to fold change and calculated enrichment of gene sets were evaluated within Reactome. Selected example pathways are denoted with arrows: FcɛRI (black), TNF receptors (orange), Interleukin 1 (green), Interleukin 12 (blue), Interferon (red), Antigen presentation (purple), MHC class I/II presentation (cyan). **c** Differentially expressed (FDR corrected) genes are shown for each selected pathway (FcɛRI; *n* = 37, *n* = 49; TNF: *n* = 39, *n* = 13; Interleukin 1: *n* = 44; Interleukin 12: *n* = 17; Interferon: *n* = 67; Antigen presentation: *n* = 29, *n* = 44, *n* = 50, *n* = 45; and MHC class I/II presentation: *n* = 42, *n* = 125, differentially expressed genes for each example pathway, respectively). Source data are provided as a Source Data file. Full statistical analyses shown in Supplementary Table 3. *p ≤ 0.05.

To gain an insight into the immune pathways associated with efficacy of IgE antibody treatment, gene expression analysis was performed on melanoma samples retrieved at the end of these in vivo experiments (Fig. 6). Enhanced expression of monocyte and macrophage gene signatures were observed in CSPG4-expressing xenografts from CSPG4 IgE-treated animals compared to controls (Fig. 6a). Enrichment of gene sets within the Reactome were ranked according to fold change (Fig. 6b). Transcriptomic analyses revealed several immune signaling pathways enriched in the IgE treatment group, including FcɛRI, TNF receptors, Interferon, Interleukins −1 and −12, antigen presentation associated pathways and MHC class I/II presentation (Fig. 6c). These findings are consistent with potential activities of monocytes and macrophages, and pro-inflammatory signals, including elevated TNF production, which were also observed with CSPG4 IgE stimulation of human monocytes ex vivo (Fig. 4c, e) and with human immune cell infiltration in melanoma xenografts of mice treated intravenously with CSPG4 IgE (Fig. 5d).

**Fig. 7:**
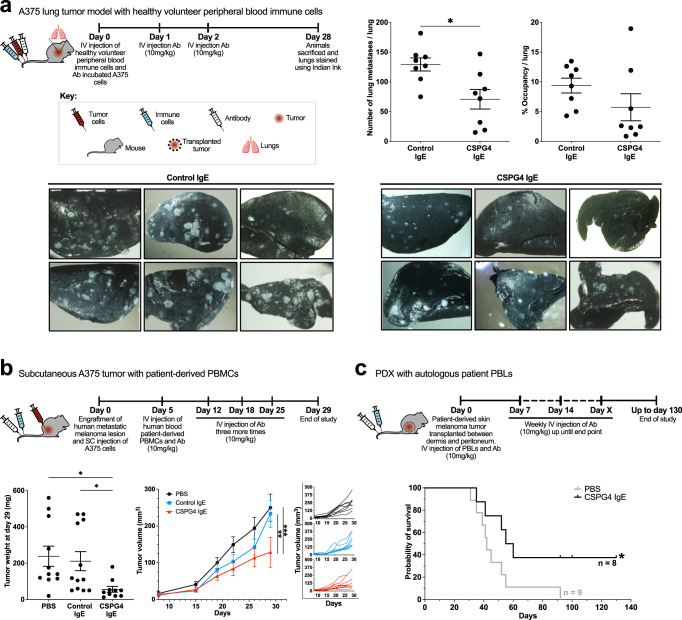
Efficacy of CSPG4 IgE in A375 and PDX tumor models. **a** Upper left: Design and dosing regimen for in vivo model. IV = intravenous, Ab = antibody. Upper right: In an A375 human melanoma model of lung metastases, engrafted with healthy volunteer peripheral blood immune cells, the number of metastases per lung (*n* = 8; *p* = 0.0107), and % occupancy by tumor metastases per lung (*n* = 8) were reduced in CSPG4 IgE-treated animals, compared to those treated with a non-specific isotype control IgE (Control IgE). Lower: Representative images of Indian ink-stained lungs showing tumor metastases in white. **b** Upper: Design and dosing regimen for in vivo model. SC = subcutaneous. Lower left: In an A375 subcutaneous model engrafted with melanoma patient-derived PBMCs, mice treated with CSPG4 IgE had significantly lower tumor weights at the end of the study, compared to those treated with Control IgE (*n* = 11, *n* = 12, *n* = 10) (*p* = 0.0243 and *p* = 0.0408). Lower right: In the same model, tumor volume was significantly lower in animals treated with CSPG4 IgE, compared to Control IgE. Inset graphs to the right show tumor growth curves for individual animals (PBS and Control IgE; *n* = 8, CSPG4 IgE; *n* = 10). Full statistical analyses shown in Supplementary Table 4 (growth curve). **c** Upper: Design and dosing regimen for in vivo model. PBLs = peripheral blood lymphocytes, PDX = patient-derived xenograft. Lower: In mice transplanted with patient-derived xenografts from two patients with stage III and IV melanoma alongside intravenous autologous patient PBLs, survival was significantly greater with CSPG4 IgE treatment compared to vehicle control (*n* = 8 and *n* = 9, respectively) (*p* = 0.0425). Data presented as mean ± SEM and Kaplan–Meier survival curves. Source data are provided as a Source Data file. Two-tailed unpaired Student’s *t* test (**a**, left), Mann–Whitney (**a**, right), One-way ANOVA (**b**, left), Two-way ANOVA (**b**, right), Log-rank Mantel–Cox test (**c**): *p ≤ 0.05; **p ≤ 0.01; ***p ≤ 0.001.

To further evaluate the effects of CSPG4 IgE in restricting melanoma dissemination, mice engrafted with healthy volunteer peripheral blood immune cells were challenged with A375 melanoma cells via the tail vein, resulting in formation of lung lesions. Lungs excised after 28 days showed a significantly lower number of melanoma lesions per lung and a trend towards lower percentage area of tumor occupancy in the lungs with CSPG4 IgE compared with control IgE (Fig. 7a). To evaluate whether CSPG4 IgE can restrict the growth of melanoma in the context of patient immune cells, mice engrafted with melanoma patient-derived immune cells were challenged with subcutaneous A375 human melanomas. Melanoma xenograft weights (29 days after tumor challenge) were significantly lower in mice given intravenous treatment with CSPG4 IgE compared with no antibody or non-specific isotype IgE control groups (Fig. 7b, left). Similarly, tumor growth was significantly restricted in mice treated with CSPG4 IgE, versus the PBS and isotype control IgE groups (Fig. 7b, right). No statistically significant differences in tumor weights, or tumor volumes, were measured between PBS and isotype control IgE-treated animals (Fig. 7b). Furthermore, to study the potential of CSPG4 IgE to restrict patient-derived melanoma growth, mice were transplanted with patient-derived cutaneous melanoma tumor xenografts (PDX) from two individuals with stage III and IV cutaneous melanoma metastases and engrafted with autologous peripheral blood lymphocytes (PBLs) from the same patients. Weekly intravenous CSPG4 IgE treatment was associated with significantly longer survival compared with control (Fig. 7c).

Therefore, across disparate in vivo models of melanoma in immunodeficient mice engrafted with healthy volunteer or melanoma patient immune cells and in PDX-bearing mice reconstituted with autologous patient immune cells, CSPG4 IgE was associated with significant tumor growth restriction or improved survival, compared to controls. Efficacy of CSPG4 IgE in vivo was associated with significant infiltration by CD68^+^ macrophages in tumors and with significant activation of Fcɛ receptor and several pro-inflammatory immune pathways at the transcriptomic level.

### In vitro and ex vivo evaluation of potential type I hypersensitivity to CSPG4 IgE

**Fig. 8:**
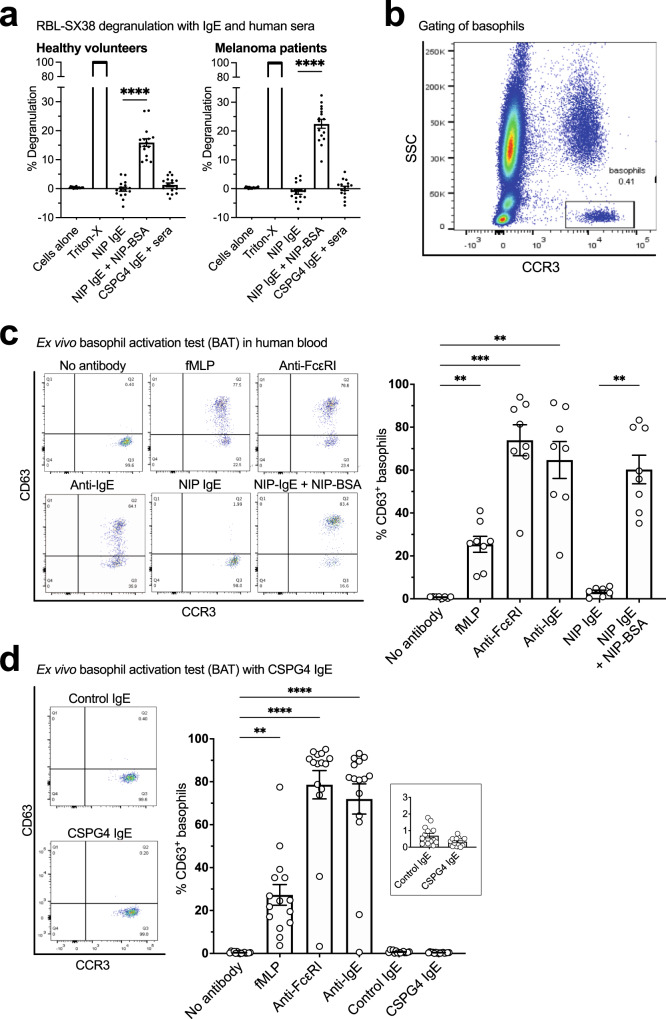
CSPG4 IgE does not mediate RBL-SX38 cell degranulation in the presence of human sera from patients or healthy participants and does not trigger basophil activation in cancer patient blood ex vivo. **a** In the absence of cancer cells, FcεRI-expressing RBL-SX38 cells sensitized with CSPG4 IgE did not degranulate when incubated with sera from healthy volunteers (left, *n* = 16) or from melanoma patients (right, *n* = 31) (p≤0.0001). **b**–**d** Basophil activation test (BAT) was performed to assess the potential risk of hypersensitivity to CSPG4 IgE treatment in human blood samples ex vivo. **b** Gating strategy to identify CCR3^high^SSC^low^ basophils in unfractionated whole blood samples. **c** Incubation of whole blood from cancer patients with positive control stimuli (fMLP, anti-FcεRI and anti-IgE), or NIP IgE and its polyclonal antigen NIP-BSA, triggered basophil activation as measured by increased CD63 cell-surface expression (representative plots, left; and summary of data from *n* = 8 independent experiments, right) (*p* = 0.0018, *p* = 0.0003, *p* = 0.0030, *p* = 0.0028). **d** CSPG4 IgE and isotype control IgE did not trigger human basophil activation in whole blood samples from melanoma patients (Left: representative plots; Right: *n* = 15 patient samples) (*p* ≤ 0.0001, *p* ≤ 0.0001, *p* = 0.0016). Inset graph shows CSPG4 IgE and Control IgE on a smaller axis scale. Data shown as mean ± SEM. Source data are provided as a Source Data file. One-way ANOVA (**a**, **c**, **d**): **p ≤ 0.01; ***p ≤ 0.001, ****p ≤ 0.0001.

To gain preliminary insights of the perceived risk of type I hypersensitivity associated with IgE, we asked whether CSPG4 IgE could potentiate degranulation of patient-derived circulating basophils. Firstly, the potential for CSPG4 IgE to trigger degranulation in the presence of healthy and patient sera was tested in the RBL-SX38 degranulation model. Whilst cross-linking of NIP IgE (positive control) by its specific multimeric antigen (NIP-BSA) triggered significant degranulation, incubation with CSPG4 IgE with sera from healthy volunteers (*n* = 16) and patients with melanoma (*n* = 15) triggered no degranulation above background (Fig. 8a). Furthermore, potential activation of primary human basophils by IgE ex vivo was studied in whole unfractionated blood from patients with melanoma using the basophil activation test (BAT) (Fig. 8b–d). This assay is employed to test potential for hypersensitivity to drugs and allergens^40,41^. Incubation of patient blood with known basophil activation stimuli; non-IgE-mediating fMLP, IgE-mediating anti-FcεRI and anti-IgE, and with NIP IgE together with NIP-BSA, triggered activation of SSC^low^CCR3^high^ basophils (measured by increased CD63 cell-surface expression) (Fig. 8c). Exogenous (CSPG4) IgE bound to basophils following 30 min incubation with unfractionated whole human blood (Supplementary Fig. 8). Neither CSGP4 IgE, nor a non-CSPG4 control IgE, triggered basophil activation when incubated ex vivo in whole blood samples from patients with melanoma (*n* = 15) (Fig. 8d). Basophil activation was not triggered by CSGP4 IgE, or by non-CSPG4 control IgE, following ex vivo stimulation of unfractionated whole blood for up to 8 hours (Supplementary Fig. 9a). Stimulation of unfractionated whole blood with intermediate to low CSPG4-expressing cells (WM1366, WM115, WM1361) together with CSPG4 IgE, or non-CSPG4 control IgE, for up to 8 hours did not activate basophils (Supplementary Fig. 9b).

CSPG4 IgE did not induce RBL-SX38 cell degranulation in the presence of patient sera and did not activate basophils in whole blood of patients with melanoma. These findings provide preliminary indications for the absence of early signals of ex vivo basophil activation with CSPG4 IgE in patient sera and blood.

## Discussion

Malignant melanoma is the most aggressive skin cancer. Despite the emergence of targeted and immune therapies, many patients do not benefit sufficiently due several resistance mechanisms. Cancer-associated antigen targets, such as chondroitin sulfate proteoglycan 4 (CSPG4), may be promising for cancer treatment^3,42^. CSPG4-specific antibodies, and engineered chimeric antigen receptor (CAR) lymphocyte therapies, have previously been shown to significantly reduce lung metastases and tumor recurrence in mouse models of melanoma^3,42–48^. Anti-CSPG4 antibodies designed with immune-activating features and able to restrict tumor growth are still required. Based on emerging pre-clinical efficacy, immune activatory functions and promising clinical studies of the first IgE class antibody specific for tumor-associated antigens^13,14,16,17,24,37^, here we designed CSPG4 IgE by combining the CSPG4-specific variable domains of a mouse clone (225.28), with a human IgE backbone.

Transcriptomic analyses confirmed higher CSPG4 gene expression in melanoma cells and tissues compared to other cancer types and normal skin tissues. Using commercially available and our own engineered CSPG4-specific antibodies, we demonstrated CSPG4 expression in malignant melanoma, including in lymph and distant metastases, and absent/low expression in normal tissues (Figs. 1, 2)^1,42,44,45^, together supporting the potential of CSPG4 as a specific tumor-associated therapeutic target. Previous studies also showed that the anti-CSPG4 225.28 antibody with high affinity (1 × 10^−9^) for CSPG4^49^, binds to a unique epitope on CSPG4 compared to other clones, and not to ubiquitously expressed carbohydrates^45,50–52^. Furthermore, clone 225.28 has been safely introduced to melanoma patients, suggesting a low risk of off-target-related toxicities^53^ for a 225.28-based treatment for melanoma.

We characterized CSPG4 IgE for its anti-tumor functional attributes (Fig. 3). In concordance with previous findings for the 225.28 mouse clone, CSPG4 IgE demonstrated partial inhibition of adhesion, migration and invasion of human melanoma cells, suggesting potential for restriction of these key mechanisms of cancer cell metastasis^42,54^. CSPG4 IgE conferred Fc-mediated functions in vitro in the presence of high and intermediate CSPG4-expressing melanoma cells: ADCC mediated by human effector cells, including monocytes from healthy individuals and patients with melanoma; and RBL-SX38 cell degranulation. No effector functions were observed in the presence of low or non-CSPG4-expressing cells (Supplementary Figs. 5, 6). These point to the requirement for target antigen specificity in order for CSPG4 IgE to potentiate Fc-mediated effector functions. Overall, these Fc-mediated functions are consistent with reports for several other tumor antigen-specific IgE antibody therapeutic candidates, such as those targeting Her2, epidermal growth factor receptor (EGFR) and CD20^14–17,24,37^.

To evaluate the in vivo efficacy of CSPG4 IgE we developed disparate animal models (Figs. 5, 7, Supplementary Fig. 7). Compared to non-specific IgE, CSPG4 IgE significantly decreased subcutaneous melanoma tumor volume and weight, and lung lesional growth, in healthy volunteer and melanoma patient-derived immune cell-engrafted mice. Efficacy of CSPG4 IgE was superior to CSPG4 IgG in the subcutaneous human melanoma model. These findings are in concordance with our studies of the first-in-class anti-tumor IgE candidate, MOv18 IgE^13^. This difference in efficacy was despite much faster clearance of IgE from the circulation in our mouse model, and in keeping with a recent in-depth study of in vivo trafficking of CSPG4 IgE and IgG antibodies in mice using SPECT imaging. In these studies, while blood clearance and hepatic accumulation of CSPG4 IgE was much faster than the counterpart IgG, tumor-to-blood and tumor-to-muscle ratios were comparable for the two isotypes^55^. Finally, in patient-derived subcutaneous melanoma xenografts engrafted with autologous immune cells from the same patient, we observed significantly longer survival of mice receiving CSPG4 IgE, compared to controls treated with the patient’s immune cells alone. Taken together, these studies demonstrated that CSPG4 IgE, in the presence of immune cells from healthy volunteers and from melanoma patients, had significant anti-melanoma activity in vivo. Efficacy was observed against tumors located in different sites; subcutaneous melanomas and melanoma lung pseudo-metastases, suggesting that anti-tumoral effects may occur at both cutaneous and lung sites. Furthermore, although no specific safety observations were made in these in vivo models, no overt toxic events were recorded. Consistently, a surrogate CSPG4 rat IgE of the clone presented in this study, that cross-reacts with the rat homolog of CSPG4, showed a favorable safety profile in immunocompetent rats^4^.

Previous studies have pointed to functions of monocytes and macrophages as effector cells, alongside activation of pro-inflammatory mediators TNF and the macrophage chemoattractant CCL-2/MCP-1, associated with IgE immunotherapy. In support of IgE-mediated immunostimulatory functions, here we demonstrated that cross-linking of CSPG4 IgE on the surface of human monocytes induced a phenotypic shift in several pro-inflammatory markers, including increased expression of co-stimulatory molecules, CD80, CD86 and CD40, simultaneous reduction of the scavenger receptor CD163 on the surface of human monocytes (Fig. 4), and enhanced levels of IL-1β, IL-6, TNF, and MCP-1. These observations, alongside the absence of IL-4 and IL-23 upregulation, associated with allergy and cytokine storm, may indicate the activation of mechanisms more akin to anti-parasitic, rather than allergic responses (Fig. 4)^56,57^. These support previous observations of the effects of MOv18 IgE antibody on ovarian cancer patient-derived monocytes^13,31,58^.

Consistent with these findings, human immune cell infiltration into subcutaneous tumors, particularly by CD68^+^ macrophages, was greater in CSPG4 IgE-treated mice compared to CSPG4 IgG and controls (Fig. 5). Subsequent transcriptomic pathway analysis of these samples revealed enrichment of monocyte and macrophage gene signatures, and activation of several immune signaling pathways including FcεRI, TNF receptors, Interferon, Interleukins 1 and 12, antigen presentation and MHC class I/II presentation in animals treated with CSPG4 IgE (Fig. 6). These are in line with previous findings in several rodent solid tumor models, suggesting that alongside prolonged survival and restricted tumor growth, IgE immunotherapy may promote macrophage infiltration and support a pro-inflammatory tumor microenvironment^11,13^. In this study, we also demonstrate that depleting PBMCs of monocytes impaired CSPG4 IgE-mediated ADCC and tumor growth restriction in vivo. Furthermore, antigen-dependent antibody-promoted interactions between monocytes and tumor cells were important for the induction of pro-inflammatory signals and CSPG4 IgE ADCC was impaired by a PTK2 inhibitor (Supplementary Fig. 6). These denote the requirement for IgE-Fc receptor signaling for the induction of immuno-activatory signals, and are consistent with previous findings that incubation with IgE antibodies increased cell-to-cell contact between monocytes and target cancer cells, an interaction abrogated by Fcɛ receptor blockade^12^. Together, our data may suggest an IgE-mediated shift towards pro-inflammatory monocyte and macrophage phenotypes and may signify reciprocal monocyte-mediated activation of anti-tumor immunity such as via T cell co-stimulation and priming, a notion supported by activation of antigen presentation pathways in the tumors of mice treated with CSPG4 IgE. Our data thus expand upon previous findings, to reveal contributions of a wider pro-inflammatory pathway signature implicating IL-1, IL-12, and Interferon and of both antigen and MHC class I/II presentation in the context of IgE therapeutics. Future studies of CSPG4 IgE, and other anti-tumor IgE candidates, may provide an insight into these pathways, the potential roles of different immune cells such as antigen-presenting cells i.e., dendritic cells and B cells, in addition to monocytes/macrophages, and their crosstalk with T cells or NK cells in the tumor microenvironment.

Overall, we demonstrated the cancer target-specific in vitro functions of CSPG4 IgE, and Fc-mediated effector activity restricted to target-expressing tumor cells, but not to low- or non-expressing normal cells. Functional assays suggest that the cross-linking of CSPG4 IgE promotes a pro-inflammatory response mediated through monocytic cells. This is evident by the release of cytokines and the upregulation of co-stimulatory cell-surface markers upon monocyte activation by CSPG4 IgE in vitro, and tumor infiltration of macrophages and immune signaling pathway activation in tumors of CSPG4 IgE-treated mice.

The main perceived risk of IgE immunotherapeutic agents is that of type I hypersensitivity and anaphylaxis which may be induced by circulating multivalent tumor antigen, shed from the tumor, and in the presence of autoantibodies. These may have the propensity to form immune complexes with CSPG4 IgE in the patient circulation, potentially resulting in cross-linking and activation of IgE Fc receptor-bearing immune cells such as basophils^26,59^. Therefore, we aimed to gain preliminary insights of the potential for type I hypersensitivity to CSPG4 IgE in ex vivo assays (Fig. 8). CSPG4 IgE did not trigger RBL-SX38 cell degranulation in the presence of sera from human healthy participants, or from patients with melanoma, despite the absence of endogenous IgEs on FcεRI in this cellular model, meaning that CSPG4 IgE could fully occupy all available Fcε receptors and to thus trigger maximum degranulation. The BAT has been increasingly applied in the cancer field to evaluate propensity for type I hypersensitivity to chemotherapy and antibody therapies in human blood ex vivo^60,61^. This assay benefits from testing basophil activation in whole unfractionated blood, which allows any potential mediators of activation in the patient circulation to be present alongside the test therapeutic agent ex vivo. We have recently employed the BAT to study basophil activation in the blood of patients with ovarian cancer^62^, and in the presence of the anti-FRα IgE (MOv18)^59^. Furthermore, the BAT has been incorporated as an eligibility criterion, and monitoring companion alongside other clinical parameters, for the phase I clinical trial of this therapeutic candidate (NCT02546921)^27^. Therefore using the BAT, here we evaluated the potential of CSPG4 IgE to trigger basophil activation. Ex vivo stimulation of whole unfractionated blood samples from melanoma patients with CSPG4 IgE did not induce basophil activation, despite clear activation by well-described IgE and non-IgE specific immune stimuli in the same samples. Taken together, our data suggest that any molecules present in the melanoma patient circulation, or intermediate-low CSPG4-expressing cells, did not have the capacity to activate basophils. However, the ex vivo BAT remains to be evaluated alongside a clinical trial.

In this study, we show that our clone does not cross-react with mouse CSPG4 antigen (Supplementary Fig. 2). Therefore, in in vivo mouse models presented herein, CSPG4 IgE selectively targets human CSPG4-expressing tumors and not any off-tumor endogenous mouse CSPG4 antigen. However, we have previously demonstrated the cross-reactivity of our antibody clone with the rat CSPG4 antigen, which we showed was minimally expressed by normal tissues with similar distribution between humans and rats^4^. In this model, repeated administration of a surrogate rat CSPG4 IgE to immunocompetent rats did not trigger overt toxicities or anaphylaxis. Although previous findings of CSPG4 IgE administration in a fully immunocompetent rat model may indicate lack of on-target off-tumor toxicity against minimally CSPG4-expressing normal tissues, the safety of CSPG4 IgE requires further investigation.

**Fig. 9:**
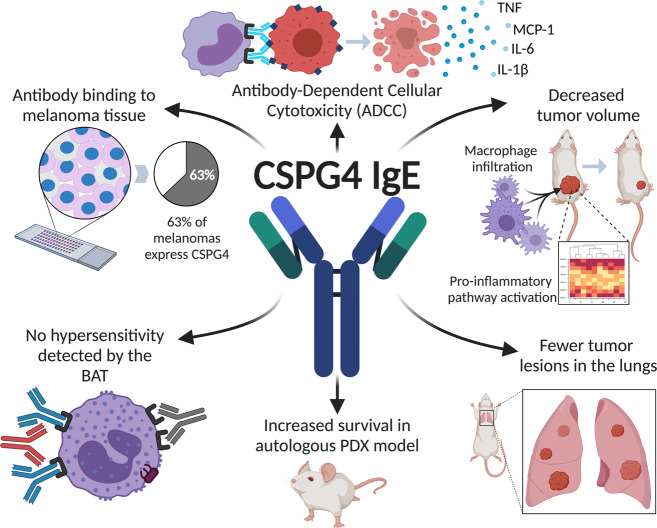
Efficacy and mechanism of action of an IgE antibody specific for the tumor-associated antigen CSPG4 to target melanoma support the development of IgE therapies for CSPG4-expressing tumors. CSPG4 IgE bound to a high proportion of melanomas, including metastases. CSPG4 IgE-mediated antibody-dependent cellular cytotoxicity (ADCC) of CSPG4-expressing melanoma cells by immune effector cells from healthy volunteers or patients with melanoma and stimulated human FcɛRI-expressing effector monocytes towards pro-inflammatory states. CSPG4 IgE restricted melanoma growth in patient-relevant in vivo models using human-derived immune cells and was associated with macrophage infiltration into tumors and activation of pro-inflammatory pathways. The antibody also prolonged the survival of mice bearing patient-derived xenografts (PDX) reconstituted with autologous immune cells from the same patient. Ex vivo basophil activation test (BAT) was used to predict that CSPG4 IgE may not induce type I hypersensitivity in melanoma patients. Created with BioRender.com.

In conclusion, in vitro studies showed that CSPG4 IgE-mediated anti-tumor activity against human melanoma cells expressing CSPG4 by immune cells derived from both healthy volunteers and melanoma patients. CSPG4 IgE treatment restricted tumor growth or improved survival across distinct in vivo models, irrespective of the tumor site, and with engraftment of immune cells from either healthy volunteers or patients (Fig. 9). We also demonstrated that CSPG4 IgE increased macrophage tumor infiltration and associated with activation of several pro-inflammatory immune pathways. These cells and pro-inflammatory signaling have previously been described pre-clinically as important for robust anti-tumor activity of IgE therapies. Finally, the ex vivo BAT provides preliminary evidence of the absence of type I hypersensitivity to CSPG4 IgE, albeit its application in a clinical setting is yet to be established. These promising findings could have far-reaching benefits for the treatment of different solid tumors, particularly those lacking approved cancer-targeting antibody therapies.

## Methods

The research was conducted at Guy’s and St. Thomas’ NHS Trust and complies with all relevant ethical regulations. The study was approved by Guy’s Research Ethics Committee and the London-Central Research Ethics Committee (REC numbers 09/H0804/45 and 16/LO/0366, respectively). Both male and female human participants were included. Written informed consent was obtained from all participants.

### Cell isolation and culture

Cells lines (human and rat effector cells and human cancer cell lines) were sourced and cultured as described in Supplementary Methods. Primary melanocytes (ATCC PCS-2000-012), human peripheral blood mononuclear cells (PBMCs), human peripheral blood lymphocytes (PBL) and purified monocytes were derived from healthy volunteer and melanoma patient blood by standard Ficoll separation (Ficoll Paque Plus; Sigma) and as described in detail in Supplementary Methods^13,31,63^.

### Immunohistochemistry/immunofluorescence

Detection and visualization of CSPG4 expression in normal and melanoma tissues, and analyses of human xenograft samples following in vivo tumor growth, are described in Supplementary Methods.

### Production and characterization of recombinant CSPG4 IgE

CSPG4 human/mouse chimeric IgE antibody, derived from clone 225.28, was engineered and produced in stable cell lines expressing anti-CSPG4 IgE^32,43,64^. Antibody purity was assessed by SDS-PAGE and SEC-HPLC as we previously described^4,14,32^. The binding of the antibody to cell-surface CSPG4 (on human tumor cell lines) and human FcεRI (on rat basophilic leukemia RBL-SX38 cells) were assessed by flow cytometry as in prior studies^13^. IgE antibodies of different specificities were used as isotype controls: NIP IgE specific for the hapten 5-iodo-4-hydroxy-3-nitrophenyl described earlier^65^. Further analyses of CSPG4 IgE specificity and cross-reactivity are described in Supplementary Methods.

### In vitro and ex vivo assays

Direct anti-tumor effects of the antibody on A375 melanoma cells, the RBL-SX38 cell degranulation assay^4,14,24^, a flow cytometric antibody-dependent cellular cytotoxicity (ADCC) and phagocytosis (ADCP) tumor cell killing assay^13,15,39^, monocyte stimulation^31^, and the basophil activation test (BAT)^59,62^ were performed as described in Supplementary Methods.

### In vivo animal models

Male and female NOD/scid/IL-2Rγ−/− mice (NOD.cg-Prkdcscid Il2rg tm1Wjl /SzJ [NSG]; The Jackson Laboratory), aged 6–10 weeks, were maintained in accordance with Institutional Committees on Animal Welfare of the UK Home Office and the Biological Services Animal Welfare & Ethical Review Body (AWERB), Guy’s Campus, King’s College London. The mice were kept on a 12 hour light/dark cycle (light of 350–400 lux). Housing conditions were maintained at 20 °C, and at a relative humidity of 40 to 60%. Malignant melanomas were established as subcutaneous tumors^63^ or injected intravenously leading to the formation of lung lesions as described in detail in Supplementary Methods. Antibody treatments were dosed at 10 mg/kg in concordance with previous in vivo studies of MOv18 IgE and SF25 IgE^13,66^. At experimental endpoints, mice were humanely euthanized using CO_2_ asphyxiation followed by cervical dislocation in accordance with The Animals (Scientific Procedures) Act 1986 (ASPA) regulated by the Parliament of the United Kingdom.

### Statistical analyses

Data are presented as mean ± Standard Error of the Mean (SEM). Statistical analyses were performed on GraphPad Prism (version 9.0). Evaluation of normal distribution of data was performed using a Shapiro–Wilk normality test. The most appropriate statistical analysis to compare data between experimental conditions was then selected (two-tailed unpaired Student’s *t* test or One-way ANOVA for normally distributed data; Mann–Whitney or Kruskal-Wallis tests for non-parametric data; and where appropriate, Two-way ANOVA). Comparisons of Kaplan-Meier survival curves were performed using a Log-rank Mantel–Cox test. Details of the statistical tests applied are included in the figure legends. Statistical analyses were performed throughout, and statistically significant differences are shown in the graphs. *p* values: *p ≤ 0.05; **p ≤ 0.01; ***p ≤ 0.001; ****p ≤ 0.0001.

### Reporting summary

Further information on research design is available in the Nature Portfolio Reporting Summary linked to this article.

## Supplementary information

Supplementary Information

Reporting Summary

## Data Availability

Source data are provided as a Source Data file. Publicly available datasets used in this study are: Cancer Cell Line Encyclopedia (CCLE; portals.broadinstitute.org/ccle); Human Protein Atlas (v20.proteinatlas.org; proteinatlas.org/ENSG00000173546-CSPG4/pathology)^29^; Gene Expression Profiling and Interactive Analyses (GEPIA; gepia.cancer-pku.cn)^67^; The Cancer Genome Atlas (TCGA; xenabrowser.net)^68^. The RNA expression datasets from transcriptomic analysis of human melanoma xenografts generated in the current study (and presented in Fig. 6, analytical methods included in Supplementary Methods) are not publicly available due to confidentiality agreements, but are available from the corresponding author upon request. Source data are provided with this paper.

## Source data

Source Data

## Code availability

Publicly available packages were used to conduct analysis. Data downloaded from The Cancer Genome Atlas (TCGA; xenabrowser.net)^68^ was cleaned and separated as detailed at github.com/rladdach/CSPG4_in_TCGA_SKCM/blob/main/TCGA_SKCM_expression_of_CSPG4.Rmd. Differentially expressed genes were identified using the package limma (3.48.3)^69^. The list of all genes, ranked according to fold change, was used to calculate enrichment of gene sets within Reactome (7.4)^70^ using package fgsea (1.18.0)^71^ with random seed set to 42. 25 gene sets of interest were visualized using ggplot2 (3.3.5)^72^. Cell specific signatures were obtained from ConsensusTME SKCM signature list (version 0.0.1.9). R version 4.1.1 was used. Fragments per kilobase million (FPKM) normalization was converted to transcripts per million (TPM) normalization and samples were stratified based on the tumor progression and site of resection or biopsy.
