# Supplementary material for: Anti-cancer pro-inflammatory effects of an IgE antibody targeting the melanoma-associated antigen chondroitin sulfate proteoglycan 4
Source: Nat Commun. 2023 Apr 25;14:2192. doi: 10.1038/s41467-023-37811-3 (PMC10130092; doi:10.1038/s41467-023-37811-3)
Supplement: Supplementary file 1 — Supplementary Information [file 41467_2023_37811_MOESM1_ESM.pdf]

SUPPLEMENTARY FIGURES

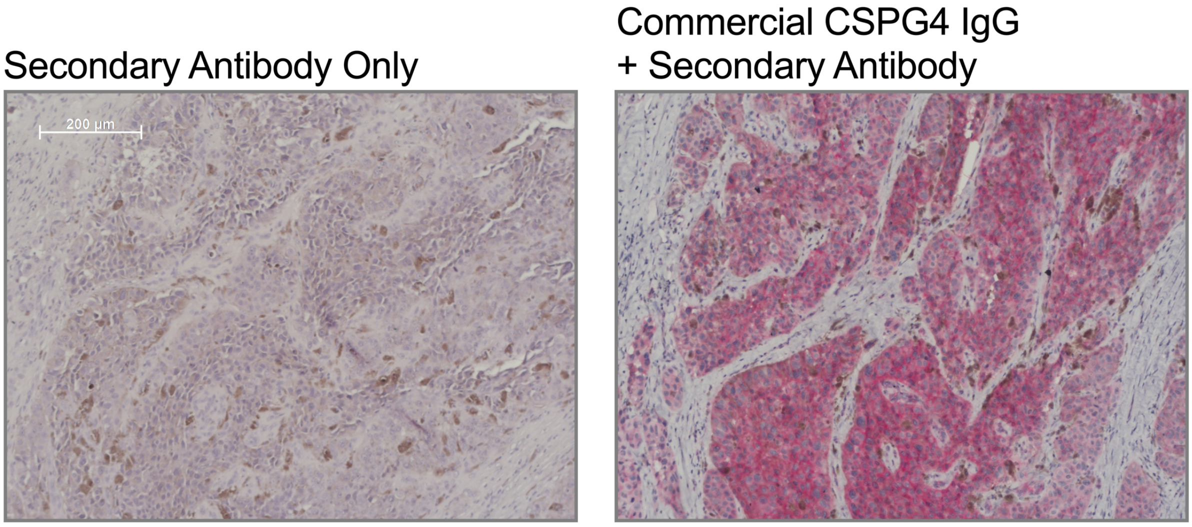

Supplementary Figure 1

Staining of commercial anti-CSPG4 IgG primary antibody or secondary antibody. Representative images of consecutive melanoma tissue sections, as in Figure 1e and 1f. Sections were stained with secondary antibody only (left) and commercial anti-CSPG4 IgG primary antibody, plus secondary antibody (right), showing no non-specific binding by the secondary antibody to melanoma tissue. Scale bar: 200µm.

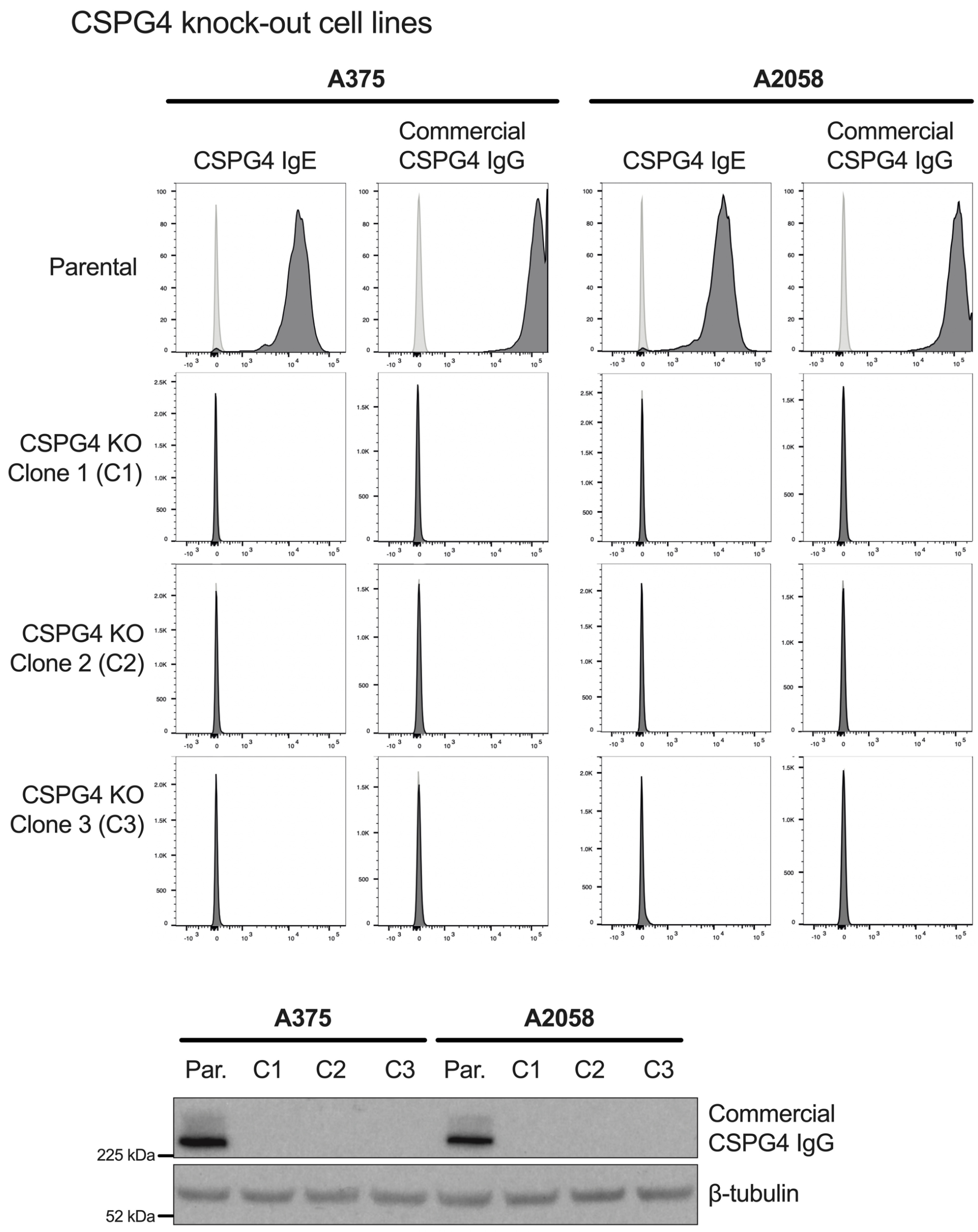

Supplementary Figure 2

CSPG4 IgE specificity to human CSPG4 antigen. The engineered CSPG4 IgE (clone 225.28) and a commercial CSPG4 IgG both bound to parental human A2058 and A375 melanoma cells, but not to 3 CSPG4 knock-out (KO) clones (C1-3) of each of these cell lines, using flow cytometry (upper). CSPG4 expression by parental human cells but not by the corresponding knock-out cells was confirmed using the commercial CSPG4 IgG antibody in Western Blot (lower).

```
sp|Q6UVK1|CSPG4_HUMAN    MQSGPRPPLPAPGLALALTLTMLARLASAASFFGENHLEVPVATALTDIDLQLQFSTSQP  60
sp|Q8VHY0|CSPG4_MOUSE    MLLGPGHPLSAPALALALTLALLVRSTAPASFFGENHLEVPVPSALTRVDLLLQFSTSQP  60
                         *  **  ** **.*******::*.* :: ************* :*** :** ********

sp|Q6UVK1|CSPG4_HUMAN    EALLLLAAGPADHLLLQLYSGRLQVRLVLGQEELRLQTPAETLLSDSIPHTVVLTVVEGW  120
sp|Q8VHY0|CSPG4_MOUSE    EALLLLAAGQDDHLLLQLHSGCLQVRLALGQKELKLQTPADTVLSDSAPHTVVLTVSDSW  120
                         *********  *******:** *****.***:**:*****:*:**** ******** :.*

sp|Q6UVK1|CSPG4_HUMAN    ATLSVDGFLNASSAVPGA-PLEVPYGLFVGGTGTLGLPYLRGTSRPLRGCLHAATLNGRS  179
sp|Q8VHY0|CSPG4_MOUSE    AVLSVDGVLNTSAPIPRASHLKATYGLFVGSSGSLDLPYLKGISRPLRGCLHSAILNGRN  180
                         *.*****.**:*: :* *  *:. ******.:*:*.****:* *********:* ****.

sp|Q6UVK1|CSPG4_HUMAN    LLRPLTPDVHEGCAEEFSASDDVALGFSGPHSLAAFPAWGTQDEGTLEFTLTTQSRQAPL  239
sp|Q8VHY0|CSPG4_MOUSE    LLRPLTSDVHEGCAEEFSAGDEVGLGFSGPHSLAAFPAWSTREEGTLEFTLTTRSQQAPL  240
                         ****** ************.*:*.***************.*::**********:*:****

sp|Q6UVK1|CSPG4_HUMAN    AFQAGGRRGDFIYVDIFEGHLRAVVEKGQGTVLLHNSVPVADGQPHEVSVHINAHRLEIS  299
sp|Q8VHY0|CSPG4_MOUSE    AFQAGDKRGNFIYVDIFEGHLRAVVEKGQGTMLLRNSVPVADGQPHEVSVHIDVHRLEIS  300
                         *****.:**:*********************:**:*****************:.******

sp|Q6UVK1|CSPG4_HUMAN    VDQYPTHTSNRGVLSYLEPRGSLLLGGLDAEASRHLQEHRLGLTPEATNASLLGCMEDLS  359
sp|Q8VHY0|CSPG4_MOUSE    VDQYPTRTFNRGVLSYLEPRGSLLLGGLDTEASRHLQEHRLGLAPGAANISLVGCIEDFS  360
                         ******:* *********************:*************:* *:* **:**:**:*

sp|Q6UVK1|CSPG4_HUMAN    VNGQRRGLREALLTRNMAAGCRLEEEEYEDDAYGHYEAFSTLAPEAWPAMELPEPCVPEP  419
sp|Q8VHY0|CSPG4_MOUSE    VNGRRQGLRDAWLTRDMSAGCRPEEDEYEEEVYGPYETFSTLAPEAWPAMELPEPCIPEP  420
                         ***:*:***:* ***:*:**** **:***::.** **:******************:***

sp|Q6UVK1|CSPG4_HUMAN    GLPPVFANFTQLLTISPLVVAEGGTAWLEWRHVQPTLDLMEAELRKSQVLFSVTRGARHG  479
sp|Q8VHY0|CSPG4_MOUSE    GLPAVFANFTQLLTISPLVVAEGGTAWLEWRHVQPTLDLTEAELRKSQVLFSVSQSARHG  480
                         *** ********************************** ************::.****

sp|Q6UVK1|CSPG4_HUMAN    ELELDIPGAQARKMFTLLDVVNRKARFIHDGSEDTSDQLVLEVSVTARVPMPSCLRRGQT  539
sp|Q8VHY0|CSPG4_MOUSE    DLELDILGAQTRKMFTLLDVVNRKARFVHDGSEDTSDQLMLEVSVTARAPVPSCLRRGQI  540
                         :***** ***:****************:***********:********.*:********

sp|Q6UVK1|CSPG4_HUMAN    YLLPIQVNPVNDPPHIIFPHGSLMVILEHTQKPLGPEVFQAYDPDSACEGLTFQVLGTSS  599
sp|Q8VHY0|CSPG4_MOUSE    YILPIQVNPVNDPPRIIFPHGSLMVILEHTQKPLGPEIFQAYDPDSACEGLTFQLLGVSS  600
                         *:************:**********************:****************:**.**

sp|Q6UVK1|CSPG4_HUMAN    GLPVERRDQPGEPATEFSCRELEAGSLVYVHRGGPAQDLTFRVSDGLQASPPATLKVVAI  659
sp|Q8VHY0|CSPG4_MOUSE    GVPVEHRDQPGEPATEFSCRELEVGDIVYVHRGGPAQDLTFRVSDGMQASAPATLKVVAV  660
                         *:***:*****************.*.:*******************:*** ********:

sp|Q6UVK1|CSPG4_HUMAN    RPAIQIHRSTGLRLAQGSAMPILPANLSVETNAVGQDVSVLFRVTGALQFGELQKQGAGG  719
sp|Q8VHY0|CSPG4_MOUSE    RPAIQILHNTGLHLAQGSAAAILPANLSVETNAVGQDVSVLFRVTGTLQFGELQKQGAGG  720
                         ****** :.***:******  ************************:************

sp|Q6UVK1|CSPG4_HUMAN    VEGAEWWATQAFHQRDVEQGRVRYLSTDPQHHAYDTVENLALEVQVGQEILSNLSFPVTI  779
sp|Q8VHY0|CSPG4_MOUSE    VEGTEWWDTLAFHQRDVEQGRVRYLSTDPQHHTQDTVEDLILEVQVGQETLSNLSFPVTI  780
                         ***:*** * **********************: ****:* ******** **********

sp|Q6UVK1|CSPG4_HUMAN    QRATVWMLRLEPLHTQNTQQETLTTAHLEATLE----EAGPSPPTFHYEVVQAPRKGNLQ  835
sp|Q8VHY0|CSPG4_MOUSE    QRATVWMLRLEPLHTQNPHQETLTPAHLEASLEEEEEEGSPQPHTFHYELVQAPRRGNLL  840
                         ***************** :***** *****:**    *..*.* *****:*****:***

sp|Q6UVK1|CSPG4_HUMAN    LQGTRLSDGQGFTQDDIQAGRVTYGATARASEAVEDTFRFRVTAPPYFSPLYTFPIHIGG  895
sp|Q8VHY0|CSPG4_MOUSE    LQGTRLSDGESFSQSDLQAGRVTYRATMRTSEAADDSFRFRVTSPPHFSPLYTFPIHIGG  900
                         *********:.*:*.*:******* ** *:***.:*:******:**:*************

sp|Q6UVK1|CSPG4_HUMAN    DPDAPVLTNVLLVVPEGGEGVLSADHLFVKSLNSASYLYEVMERPRHGRLAWRGTQDKTT  955
sp|Q8VHY0|CSPG4_MOUSE    DPNAPVLTNVLLMVPEGGEGVLSADHLFVKSLNSASYLYEVMEQPHHGKLAWRDPKGKST  960
                         **:*********:*******************************:*:**:****. :.*:*

sp|Q6UVK1|CSPG4_HUMAN    MVTSFTNEDLLRGRLVYQHDDSETTEDDIPFVATRQGESSGDMAWEEVRGVFRVAIQPVN  1015
sp|Q8VHY0|CSPG4_MOUSE    PVTSFTNEDLLHGRLVYQHDDSETIEDDIPFVATRQGEGSGDMAWEEVRGVFRVAIQPVN  1020
                          **********:************ *************.********************

sp|Q6UVK1|CSPG4_HUMAN    DHAPVQTISRIFHVARGGRRLLTTDDVAFSDADSGFADAQLVLTRKDLLFGSIVAVDEPT  1075
sp|Q8VHY0|CSPG4_MOUSE    DHAPVQTISRVFHVARGGQRLLTTDDVAFSDADSGFSDAQLVLTRKDLLFGSIVAMEEPT  1080
                         **********:*******:******************:********************::***

sp|Q6UVK1|CSPG4_HUMAN    RPIYRFTQEDLRKRRVLFVHSGADRGWIQLQVSDGQHQATALLEVQASEPYLRVANGSSL  1135
sp|Q8VHY0|CSPG4_MOUSE    RPIYRFTQEDLRKKQVLFVHSGADHGWLQLQVSDGQHQATAMLEVQASEPYLHVANSSSL  1140
                         *************::*********:**:*************:**********:***.***

sp|Q6UVK1|CSPG4_HUMAN    VVPQGGQGTIDTAVLHLDTNLDIRSGDEVHYHVTAGPRWGQLVRAGQPATAFSQQDLLDG  1195
sp|Q8VHY0|CSPG4_MOUSE    VVPQGGQGTIDTAVLQLDTNLDIRSGNEVHYHVTAGPQWGQLLRDGQSVTSFSQRDLLDG  1200
                         ***************:**********:**********:****:* **  .*:***:*****

sp|Q6UVK1|CSPG4_HUMAN    AVLYSHNGSLSPRDTMAFSVEAGPVHTDATLQVTIALEGPLAPLKLVRHKKIYVFQGEAA  1255
sp|Q8VHY0|CSPG4_MOUSE    AILYSHNGSLSPQDTLAFSVAAGPVHTNTFLQVTIALEGPLAPLQLVQHKKIYVFQGEAA  1260
                         *:*********:**:**** ******:: *************:**:************

sp|Q6UVK1|CSPG4_HUMAN    EIRRDQLEAAQEAVPPADIVFSVKSPPSAGYLVMVSRGALADEPPSLDPVQSFSQEAVDT  1315
sp|Q8VHY0|CSPG4_MOUSE    EIRRDQLEVVQEAVLPADIMFSLRSPPNAGYLVMVSHGASAEEPPSLDPVQSFSQEAVNS  1320
                         ********..**** ****:**::***.********:** *:****************::

sp|Q6UVK1|CSPG4_HUMAN    GRVLYLHSRPEAWSDAFSLDVASGLGAPLEGVLVELEVLPAAIPLEAQNFSVPEGGSLTL  1375
sp|Q8VHY0|CSPG4_MOUSE    GRVLYLHSRPGAWSDSFSLDVASGLGDPLEGISVELEVLPTVIPLDVQNFSVPEGGTRTL  1380
                         ********** ****:********** ****: *******:.***:.*********: **

sp|Q6UVK1|CSPG4_HUMAN    APPLLRVSGPYFPTLLGLSLQVLEPPQHGALQKEDGPQARTLSAFSWRMVEEQLIRYVHD  1435
sp|Q8VHY0|CSPG4_MOUSE    APPLVQITGPYFPTLPGLVLQVLEPPQHGALQKEDHSQDGSLSTFSWREVEEQLIRYVHD  1440
                         ****::::******* ** ***************  *  :**:**** ***********

sp|Q6UVK1|CSPG4_HUMAN    GSETLTDSFVLMANASEMDRQSHPVAFTVTVLPVNDQPPILTTNTGLQMWEGATAPIPAE  1495
sp|Q8VHY0|CSPG4_MOUSE    GSETQTDAFVLLANASEMDRQSQPVAFTITILPVNDQPPVLTTNTGLQIWEGAIVPIPPE  1500
                         **** **:***:**********:*****:*:*********:********:****  .*** *

sp|Q6UVK1|CSPG4_HUMAN    ALRSTDGDSGSEDLVYTIEQPSNGRVVLRGAPGTEVRSFTQAQLDGGLVLFSHRGTLDGG  1555
sp|Q8VHY0|CSPG4_MOUSE    ALRGTDNDSGPEDLVYTIEQPSNGRIALRVAPDTEVHRFTQAQLDSGLVLFSHRGALEGG  1560
                         ***.**.*** *************:.** **.***: *******.*********:*:**

sp|Q6UVK1|CSPG4_HUMAN    FRFRLSDGEHTSPGHFFRVTAQKQVLLSLKGSQTLTVCPGSVQPLSSQTLRASSSAGTDP  1615
sp|Q8VHY0|CSPG4_MOUSE    FHFDLSDGAHTSPGHFFRVVAQKQALLSLEGTRKLTVCPESVQPLSSQSLSASSSTGADP  1620
                         *:* **** **********.****.****:*::.***** ********:* ****:*:**

sp|Q6UVK1|CSPG4_HUMAN    QLLLYRVVRGPQLGRLFHAQQDSTGEALVNFTQAEVYAGNILYEHEMPPEPFWEAHDTLE  1675
sp|Q8VHY0|CSPG4_MOUSE    RHLLYRVVRGPQLGRLLHAQQGSAEEVLVNFTQAEVNAGNILYEHEMSSEPFWEAHDTIG  1680
                         : *************:****.*: *.********* **********  *********:

sp|Q6UVK1|CSPG4_HUMAN    LQLSSPPARDVAATLAVAVSFEAACPQRPSHLWKNKGLWVPEGQRARITVAALDASNLLA  1735
sp|Q8VHY0|CSPG4_MOUSE    LLLSSPPARDLAATLAVMVSFDAACPQRPSRLWKNKGLWVPEGQRAKITVAALDAANLLA  1740
                         * ********:****** ***:*********:***************:*********:****

sp|Q6UVK1|CSPG4_HUMAN    SVPSPQRSEHDVLFQVTQFPSRGQLLVSEEPLHAGQPHFLQSQLAAGQLVYAHGGGGTQQ  1795
sp|Q8VHY0|CSPG4_MOUSE    SVPASQRSRHDVLFQVTQFPTRGQLLVSEEPLHARRPYFLQSELAAGQLVYAHGGGGTQQ  1800
                         ***: ***.***********:************* :
```

Percent Identity Matrix - created by Clustal2.1

1: sp Q6UK1 CSPG4 HUMAN	100.00	83.55
2: sp Q8VHYO CSPG4_MOUSE	83.55	100.00

Supplementary Figure 3

Amino acid sequence alignment between human and mouse CSPG4. Analysis shows a 83.55% sequence similarilty. Data were generated using EMBL-EBI Clustal Omega (www.ebi.ac.uk/Tools/msa/clustalo/)[1,2].

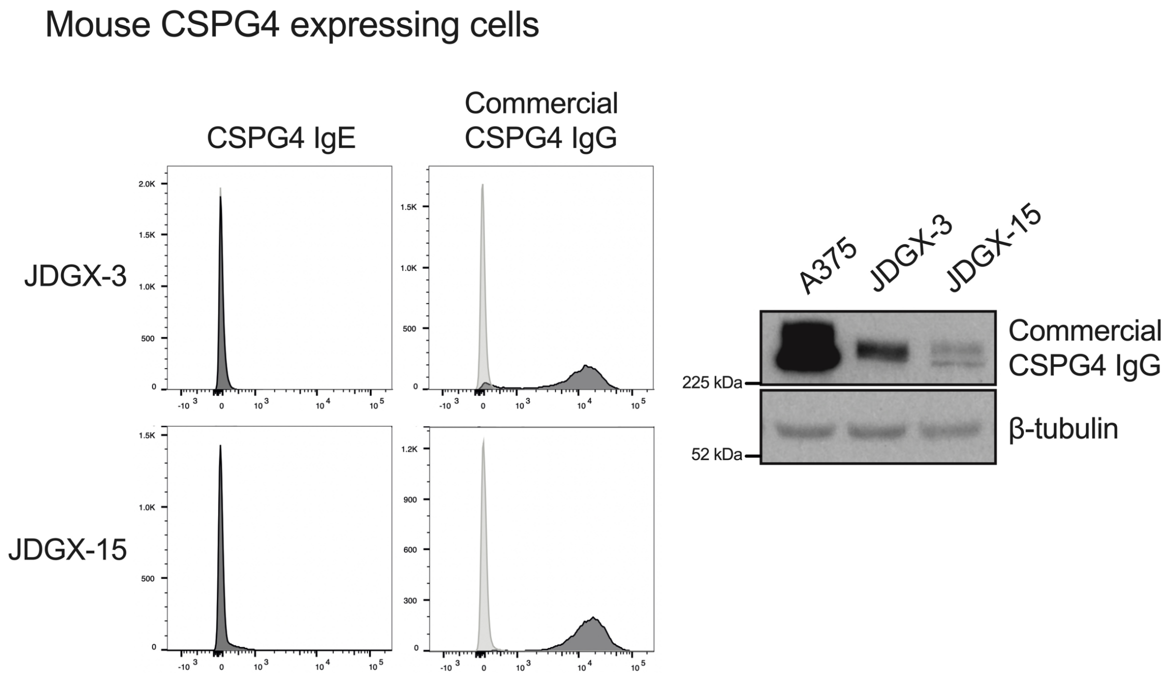

Supplementary Figure 4

CSPG4 IgE does not cross-react with mouse CSPG4. Expression of the mouse CSPG4 antigen by mouse tumor-derived melanoma cell lines obtained from the tamoxifen-inducible TyrCreER(t2)/Rac1(P29S)-LSL/BRaf(V600E)-Loxp/Trp53fl mouse model (JDGX strain) was confirmed using a commercial anti-mouse CSPG4 IgG (REA989) tested by flow cytometry and Western Blot. However, CSPG4 IgE (clone 225.28) did not bind mouse melanoma cells, as tested by flow cytometry and Western Blot analyses, indicating no cross-reactivity of the 225.28 clone with the mouse CSPG4.

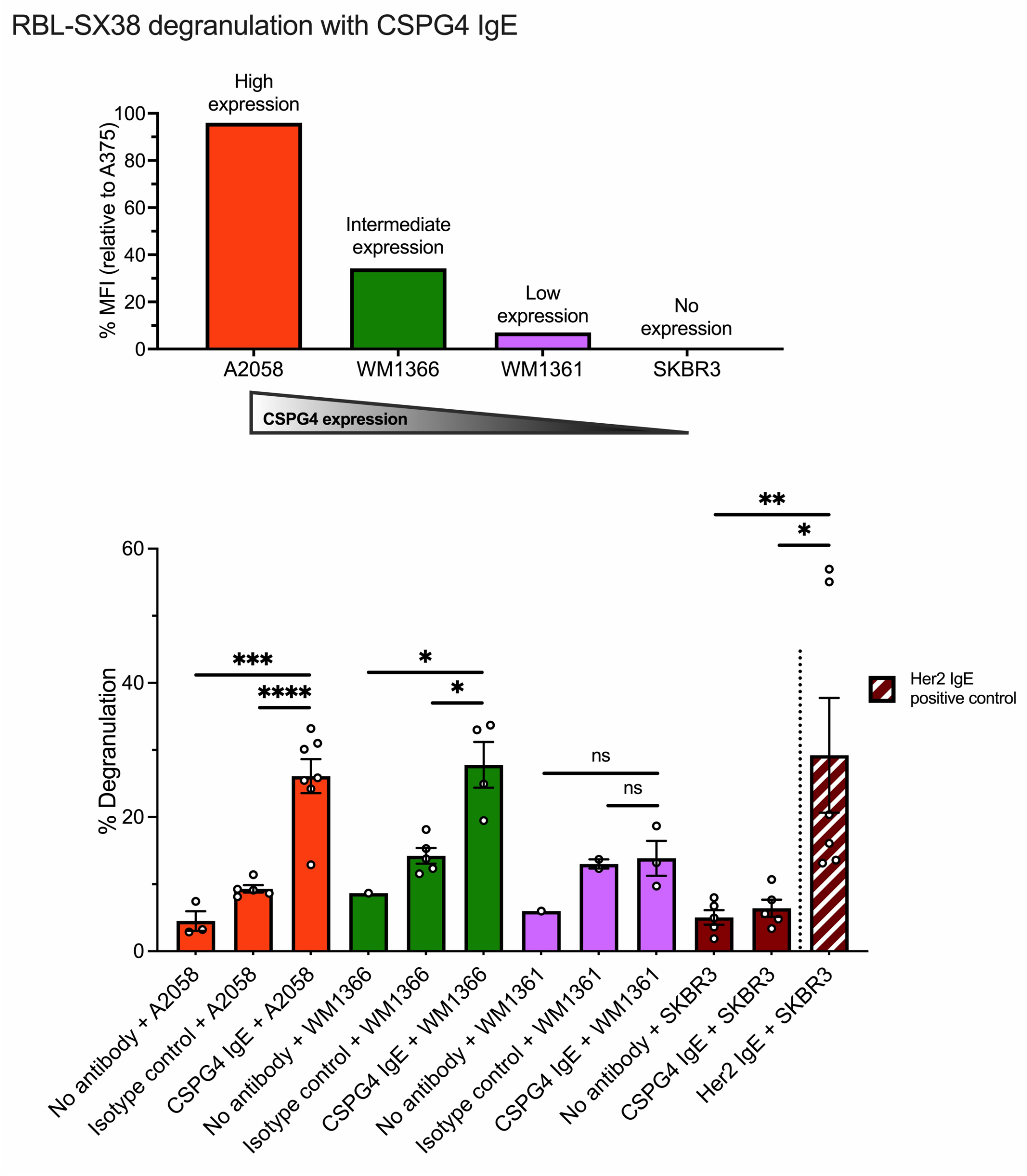

Supplementary Figure 5

RBL-SX38 degranulation by CSPG4 IgE stimulation in the presence of cancer cells is dependent on the level of CSPG4 antigen expression by tumor cells. CSPG4 IgE triggered RBL-SX38 cell degranulation, above isotype (NIP IgE) or no antibody controls, when cross-linked by A2058 and WM1366 melanoma cells, which express high and intermediate levels of CSPG4 (A2058: n=3, n=5, and n=7; $p=0.0002$, $p\leq0.0001$; WM1366: n=1, n=5, and n=4; $p=0.0198$ and $p=0.0130$, respectively. CSPG4 IgE did not trigger significant levels of RBL-SX38 degranulation when cross-linked by WM1361 and SKBR3 cells, which show low and no

expression of CSPG4, respectively. Degranulation was mediated by Her2 IgE when cross-linked by SKBR3 (a positive control for these high Her2-expressing cells, shown with a fill) (WM1361: n=1, n=2, and n=3; SKBR3: n=5, n=5, and n=6; p=0.0096 and p=0.0278, respectively). Top: Flow cytometric analyses of CSPG4 expression in different cell lines (% MFI). Bottom: % RBL-SX38 cell degranulation; Data shown as mean $\pm$ SEM; Kruskal-Wallis: * $p \leq 0.05$; ** $p \leq 0.01$; *** $p \leq 0.001$; **** $p \leq 0.0001$. Source data are provided as a Source Data file.

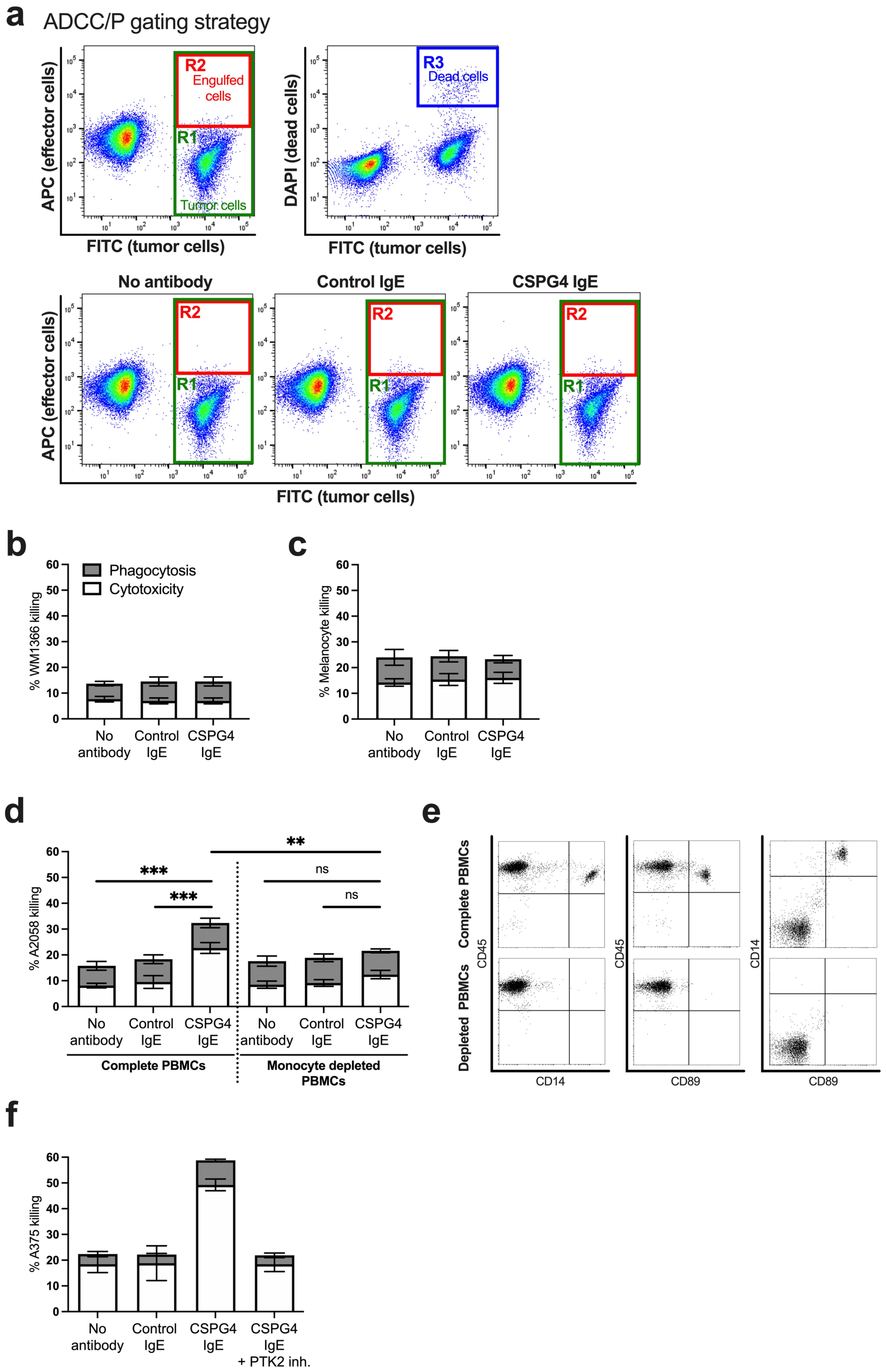
a
ADCC/P gating strategy
R2
Engulfed cells
R1
Tumor cells
APC (effector cells)
FITC (tumor cells)
R3
Dead cells
DAPI (dead cells)
No antibody
Control IgE
CSPG4 IgE
b
Phagocytosis
Cytotoxicity
% WM1366 killing
No antibody
Control IgE
CSPG4 IgE
c
% Melanocyte killing
d
% A2058 killing
***
**
ns
Complete PBMCs
Monocyte depleted PBMCs
e
Complete PBMCs
Depleted PBMCs
CD45
CD14
CD89
f
% A375 killing
CSPG4 IgE + PTK2 inh.

Supplementary Figure 6

No killing of low CSPG4-expressing and non-expressing cells by CSPG4 IgE. (**a**) Representative flow cytometric ADCC/ADCP assay plots: R1: total CFSE$^+$ tumor cell population; R2: CSFE$^+$/APC$^+$ phagocytosed cells; R3: CSFE$^+$/DAPI$^+$ dead tumor cells. (**b-c**) No cytotoxicity (white bars) or phagocytosis (grey bars) of a low CSPG4-expressing melanoma cell line (WM1366, **b**), or of non-CSPG4 expressing non-malignant human melanocytes (**c**), were mediated by CSPG4 IgE, in co-cultures with healthy volunteer or patient-derived PBMCs, respectively (WM1366 cells: No antibody, n=4; Control IgE, n=2; CSPG4 IgE, n=2; Melanocytes: n=5). (**d**) Cytotoxicity (white bars) of high CSPG4-expressing A2058 melanoma cells was mediated by CSPG4 IgE together with complete PBMCs (left), but not by CSPG4 IgE together with monocyte depleted PBMCs (right) (n=4). Complete PBMCs: p=0.0002 and p=0.0004, respectively. Complete PBMCs with CSPG4 IgE compared to monocyte depleted PBMCs with CSPG4 IgE: p=0.0054. (**e**) Representative flow cytometric dot plots showing loss of CD45$^+$/CD14$^+$, CD45$^+$/CD89$^+$, CD14$^+$/CD89$^+$ cells following monocyte depletion from PBMCs. (**f**) CSPG4 IgE mediated cytotoxicity of A2058 melanoma cells by human primary monocytes was impaired by a PTK2 inhibitor (technical replicates from one representative experimental set-up shown). Data shown as mean $\pm$ SEM. Source data are provided as a Source Data file. Kruskal-Wallis (b, c), One-way ANOVA (d). ** $p \leq 0.01$; *** $p \leq 0.001$.

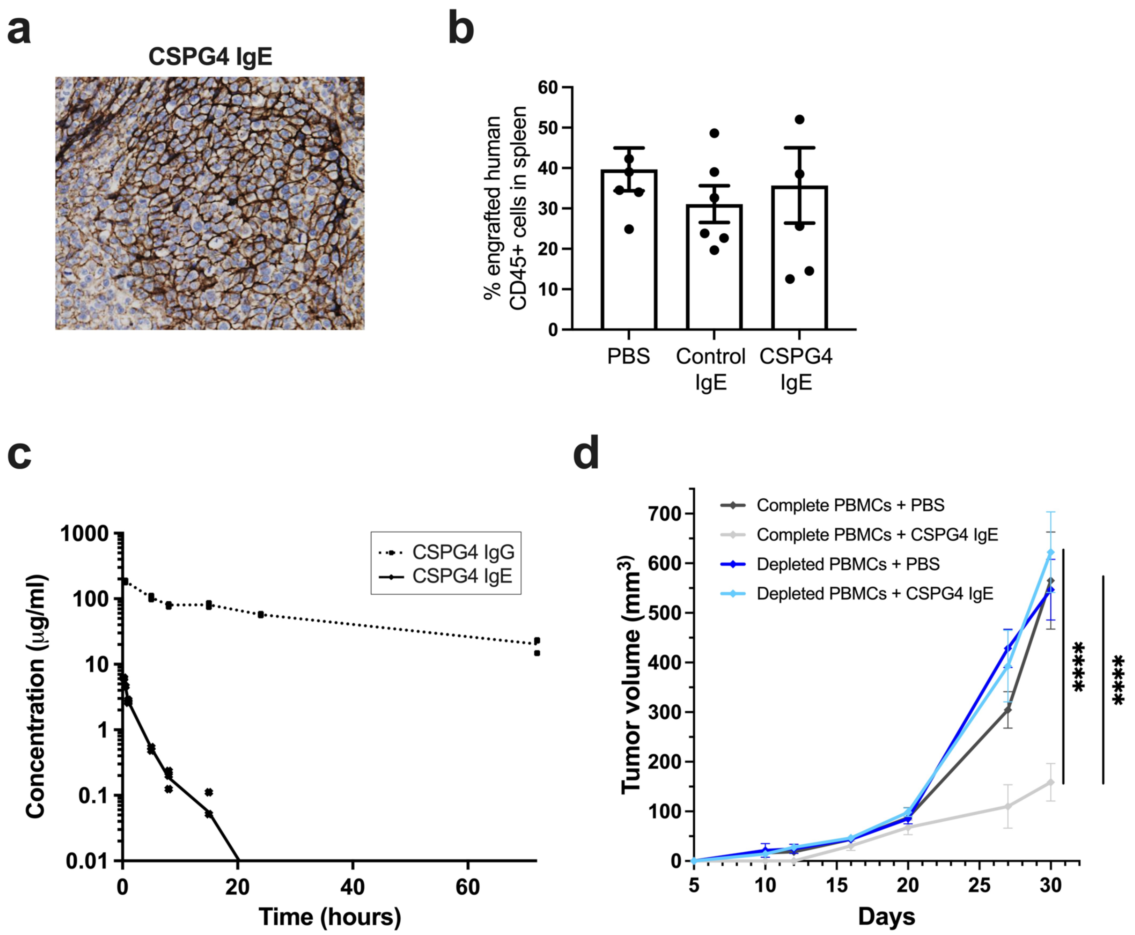

Supplementary Figure 7

Subcutaneous A375 *in vivo* model expressing CSPG4, engrafted with human CD45$^+$ immune cells in mouse spleen, clearance of CSPG4 antibodies, and ablation of CSPG4 IgE tumor growth restriction by depletion of monocytes from immune cell engraftment. (**a**) CSPG4 cell surface expression was detected on excised A375 human subcutaneous tumor xenografts by IHC using biotinylated CSPG4 IgE, detected by horseradish peroxidase (HRP, brown), and nuclei were stained with hematoxylin (blue). (**b**) % engraftment of human CD45$^+$ immune cells in mouse spleens of mice from different treatment groups at the end of the study (day 28) (n=6). (**c**) Clearance of CSPG4 IgE from the circulation was markedly faster than that measured for CSPG4 IgG (n=3). (**d**) Inhibition of subcutaneous A375 tumor growth by CSPG4 IgE was ablated by depletion of monocytes from PBMCs prior to engraftment in immunodeficient mice. Mice challenged with subcutaneous melanomas were either engrafted with complete PBMCs and treated with PBS control alone (dark grey) or CSPG4 IgE (light grey), or engrafted

with depleted PBMCs and treated with PBS alone (dark blue) or CSPG4 IgE (light blue) (PBS or antibody treatment were administered every 14 days; complete PBMCs with PBS, n=8; complete PBMCs with CSPG4 IgE, n=5; depleted PBMCs with PBS, n=6; depleted PBMCs with CSPG4 IgE, n=6 mice per group). Data shown as mean $\pm$ SEM. Source data are provided as a Source Data file. Two-way ANOVA between complete PBMCs with PBS and complete PBMCs with CSPG4 IgE, complete PBMCs with CSPG4 IgE and depleted PBMCs with CSPG4 IgE (d): **** $p \leq 0.0001$. Full statistical analyses are shown in Supplementary Table 5.

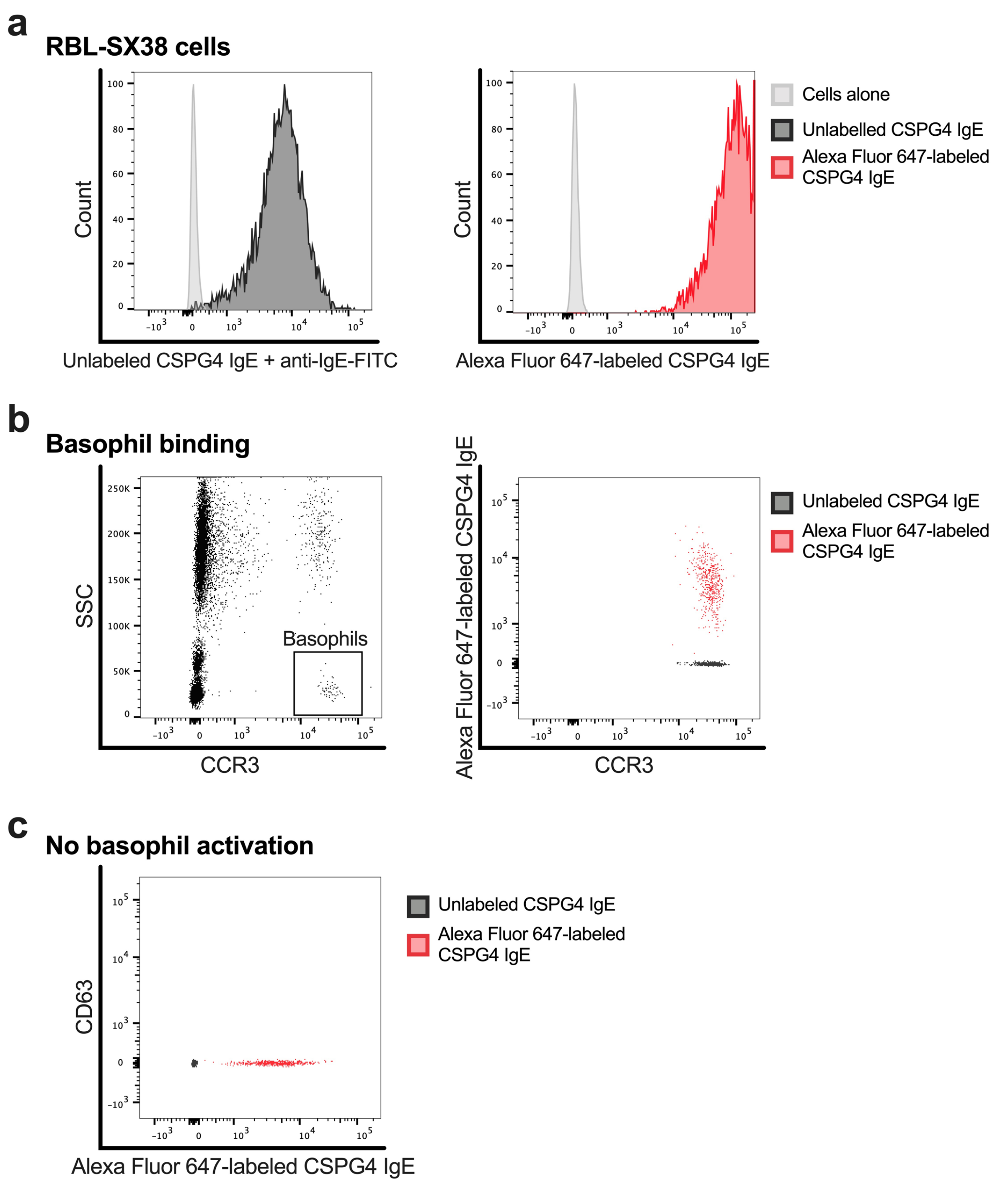

Supplementary Figure 8

Exogenous (CSPG4) IgE bound to basophils in whole unfractionated blood following incubation for 30 minutes at 37°C but did not trigger basophil activation. (**a**) Unlabeled CSPG4 IgE (detected by anti-IgE-FITC antibody) and CSPG4 IgE directly labelled with Alexa Fluor 647 bound to FcεRI-expressing RBL-SX38 cells. (**b**) CCR3-PE^{high}/SSC^{low} basophils

(identified as in flow cytometric dot plot, left) were bound by both unlabeled and Alexa Fluor 647-labeled CSPG4 IgE antibodies in whole unfractionated blood following incubation for 30 minutes at 37°C *ex vivo*. (**c**) Binding of unlabeled or Alexa Fluor 647-labeled CSPG4 IgE to CCR3-PE^{high} basophils in whole blood, following *ex vivo* incubation for 30 minutes at 37°C, did not trigger basophil activation, as measured by CD63-FITC.

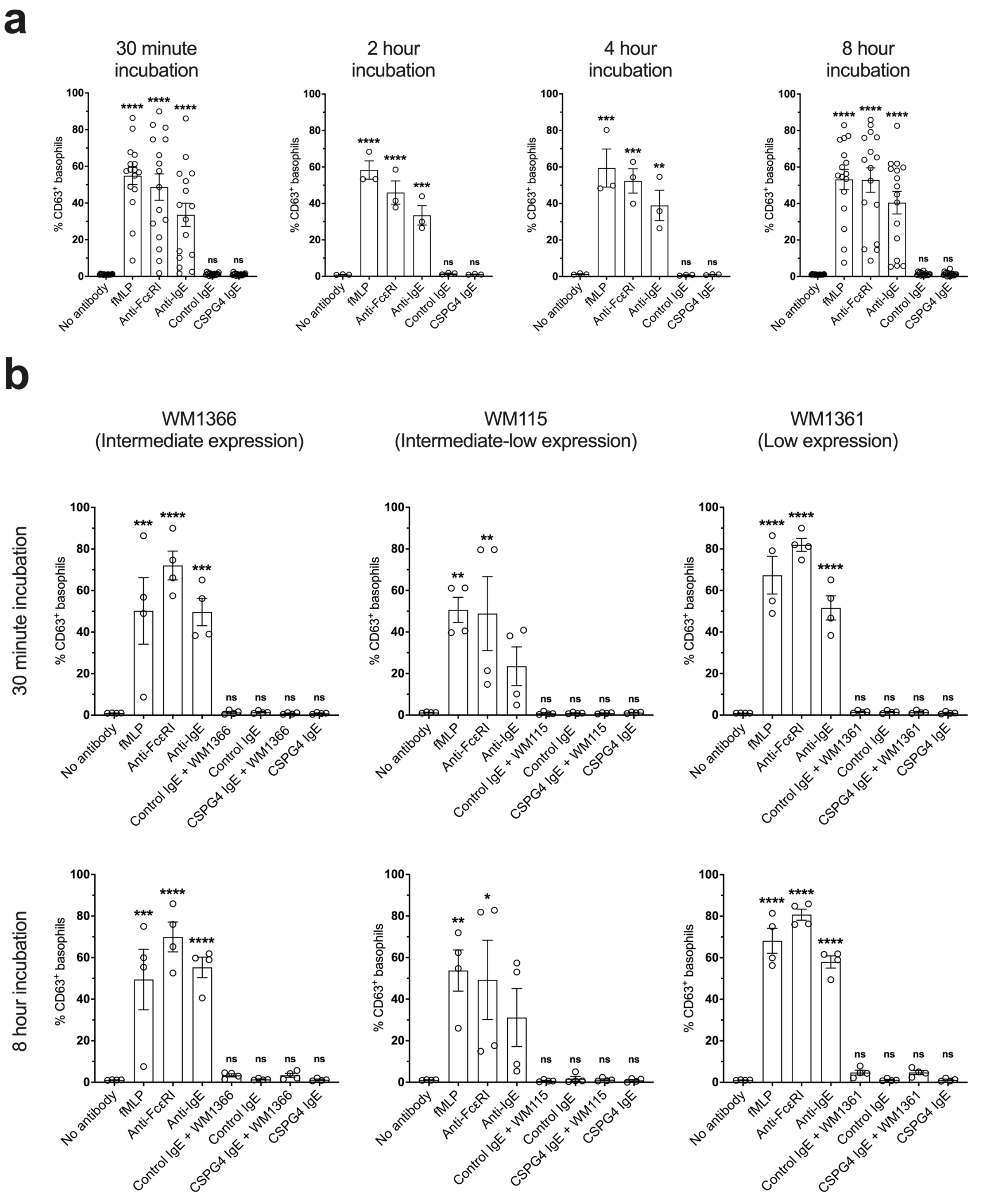

Supplementary Figure 9

***Ex vivo* stimulation of unfractionated whole blood for up to 8 hours, with CSPG4 IgE, and in the presence of low CSPG4-expressing cells, did not trigger basophil activation.** (**a**) *Ex vivo* stimulation of whole unfractionated blood with control IgE or CSPG4 IgE for 30 minutes, 2, 4

or 8 hours did not trigger basophil activation (30 minute: n=16, p≤0.0001; 2 hour: n=3, p≤0.0001, p≤0.0001, and p=0.0008; 4 hour: n=3, p=0.0003, p=0.0008, and p=0.0073; 8 hour: n=16, p≤0.0001, respectively). (**b**) In whole unfractionated blood, no basophil activation was triggered by *ex vivo* stimulation, for 30 minutes or 8 hours, with CSPG4 IgE in the presence of intermediate (WM1366), intermediate-low (WM115) or low (WM1361) CSPG4-expressing cells (n=4) (30 minutes incubation: WM1366 – p=0.0005, p≤0.0001, and p=0.0005; WM115 – p=0.0019 and p=0.0029; and WM1361 – p≤0.0001. 8 hour incubation: WM1366 – p=0.0001, p≤0.0001, and p≤0.0001; WM115 – p=0.0082 and p=0.0185; WM1361 – p≤0.0001). Data shown as mean ± SEM. Source data are provided as a Source Data file. One-way ANOVA: * p ≤ 0.05; ** p≤0.01; *** p≤0.001; **** p≤0.0001, comparison to no antibody control.

SUPPLEMENTARY TABLES

Supplementary Table 1 - 7 day interval treatment

Days	PBS vs CSPG4 IgG	PBS vs CSPG4 IgE	Control IgG vs CSPG4 IgG	Control IgE vs CSPG4 IgE	CSPG4 IgG vs CSPG4 IgE
**16**	ns	ns	ns	ns	ns
**18**	ns	ns	ns	ns	ns
**20**	ns	* (p=0.0252)	ns	ns	ns
**24**	* (p=0.0128)	** (p=0.0015)	** (p=0.0078)	* (p=0.0144)	ns
**27**	**** (p≤0.0001)	**** (p≤0.0001)	* (p=0.0240)	**** (p≤0.0001)	ns
**30**	**** (p≤0.0001)	**** (p≤0.0001)	ns	**** (p≤0.0001)	*** (p=0.0005)

Statistical test: Two-way ANOVA

Supplementary Table 2 - 14 day interval treatment

Days	PBS vs CSPG4 IgG	PBS vs CSPG4 IgE	Control IgG vs CSPG4 IgG	Control IgE vs CSPG4 IgE	CSPG4 IgG vs CSPG4 IgE
**16**	ns	ns	ns	ns	ns
**20**	ns	**	ns	ns	ns
**27**	***(p=0.0007)	**** (p≤0.0001)	ns	**** (p≤0.0001)	*** (p=0.0003)
**30**	** (p=0.0017)	**** (p≤0.0001)	ns	**** (p≤0.0001)	*** (p=0.0001)

Statistical test: Two-way ANOVA

Supplementary Table 3 - Enriched transcriptomic signaling pathways

Signaling pathway	Gene	P value (p adjusted)
**FcεRI**		
FcεRI mediated NF-κB activation	*PSMB6*<br>*PSMB10*<br>*PSMA6*<br>*PSMB2*	0.02015<br>0.02052<br>0.03051<br>0.01237
Fcε receptor FcεRI signaling	*PSMB6*<br>*PSMB10*<br>*PSMA6*<br>*PSMB2*<br>*ITPR3*	0.02015<br>0.02052<br>0.03051<br>0.01237<br>0.01267
**TNF receptor**		
TNFR2 non canonical NF-κB:	*PSMB6*<br>*PSMB10*<br>*PSMA6*<br>*PSMB2*<br>*TNFRSF12A*	0.02015<br>0.02052<br>0.03051<br>0.01237<br>0.03708
**Interleukin 1**		
Interleukin 1 signaling	*PSMB6*<br>*PSMB10*<br>*PSMA6*<br>*PSMB2*<br>*IRAK1*	0.02015<br>0.02052<br>0.03051<br>0.01237<br>0.04208
**Interleukin 12**		
Interleukin 12 signaling	*SOD1*<br>*MTAP*<br>*AIP*	0.01379<br>0.03928<br>0.04374
**Interferon**		
Interferon signaling	*CD44*<br>*HLA-B*<br>*NUP37*<br>*ISG20*<br>*EGR1*<br>*UBE2L6*<br>*IRF9*	0.01910<br>0.04208<br>0.01775<br>0.02895<br>0.01775<br>0.02895<br>0.02052
**Antigen presentation**		
Cross presentation of soluble exogenous antigens endosomes	*PSMB6*<br>*PSMB10*<br>*PSMA6*<br>*PSMB2*	0.02015<br>0.02052<br>0.03051<br>0.01237
APC-C mediated degradation of cell cycle proteins	*PSMB6*<br>*PSMB10*<br>*PSMA6*<br>*PSMB2*<br>*SKP2*	0.02015<br>0.02052<br>0.03051<br>0.01237<br>0.03123
Antigen presentation cross presentation	*PSMB6*<br>*PSMB10*<br>*PSMA6*<br>*PSMB2*<br>*HLA-B*	0.02015<br>0.02052<br>0.03051<br>0.01237<br>0.04208
Antiviral mechanism by IFN stimulated genes	*NUP37*<br>*UBE2L6*	0.01775<br>0.02895
**MHC class I/II presentation**		
Class I MHC mediated antigen processing presentation	*PSMB6*<br>*PSMB10*<br>*PSMA6*<br>*PSMB2*<br>*HLA-B*<br>*UBE2L6*<br>*SKP2*	0.02015<br>0.02052<br>0.03051<br>0.01237<br>0.04208<br>0.02895<br>0.03123

Supplementary Table 4 - Subcutaneous A375 tumor with patient-derived PBMCs

Days	PBS vs Control IgE	PBS vs CSPG4 IgE	Control IgE vs CSPG4 IgE
8	ns	ns	ns
15	ns	ns	ns
19	ns	ns	ns
22	ns	ns	ns
26	ns	* (p=0.0148)	ns
29	ns	*** (p=0.0005)	** (p=0.0037)

Statistical test: Two-way ANOVA

Supplementary Table 5 - Monocyte depleted PBMCs

Days	Complete PBMCs + PBS vs Complete PBMCs + CSPG4 IgE	Complete PBMCs + PBS vs Depleted PBMCs + CSPG4 IgE	Complete PBMCs + PBS vs Depleted PBMCs + PBS	Complete PBMCs + CSPG4 IgE vs Depleted PBMCs + CSPG4 IgE
10	ns	ns	ns	ns
12	ns	ns	ns	ns
16	ns	ns	ns	ns
20	ns	ns	ns	ns
27	ns	ns	ns	** (p=0.0020)
30	**** ($p \leq 0.0001$)	ns	ns	**** ($p \leq 0.0001$)

Statistical test: Two-way ANOVA

SUPPLEMENTARY METHODS

CSPG4 expression in cancer cell lines and tissues

CSPG4 mRNA expression was studied in a number of cancer cell types using Cancer Cell Line Encyclopedia (CCLE) (portals.broadinstitute.org/ccle). Gene expression in tissues across cancer types was studied using The Human Protein Atlas online tool (https://www.proteinatlas.org/ENSG00000173546-CSPG4/pathology).[3,4] Gene expression was also compared between normal skin and cutaneous melanoma tissues (format: expression boxplot; gene: CSPG4; dataset: SKCM; p-value cutoff: 0.05; log scale: yes; jitter size: 0.4; matched TCGA normal and GTEx data), and across stages of melanoma (format: expression pathological stage plot; gene: CSPG4; dataset: SKCM; log scale: yes; use major stages: yes) using the Gene Expression Profiling Interactive Analysis (GEPIA) online tool (http://gepia.cancer-pku.cn/index.html, analysis completed in February 2022 [Figure 1c] and July 2022 [Figure 1d right]).[5] Furthermore, FPKM normalized gene expression RNA-seq TCGA-SKCM data was obtained from xenabrowser.net.[6] FPKM normalization was converted to transcript per million (TPM) normalization and samples were stratified based on the tumor progression and site of resection or biopsy into four categories: primary tumor (n=103), metastatic skin (n=116), metastatic viscera (n=36) and metastatic lymph (n=208). Kruskal-Wallis was performed using all four categories.

Cell culture

The U937 human monocytic cell line (ATCC CRL-1593.2),[7] and the malignant melanoma cell lines A2058 (ATCC CRL-11147), A375 (ATCC CRL-1619), WM1366 (CVCL_6789), WM115 (CVCL_0040), WM1361 (CVCL_6788), G361 (ATCC CRL-1424) and SKMEL28 (ATCC HTB-72), the SKBR3 (ATCC HTB-30) breast cancer cell line and the IGROV1 (CVCL_1304) ovarian cancer cell line, were all cultured in RPMI 1640 medium, supplemented with 10% FCS, penicillin (5,000 U/ml), streptomycin (100 μg/ml) and maintained at 5% CO_2, 37°C. The RBL-SX38 rat basophilic leukemia cells, which express both the native rat FcεRI and the human tetrameric

form of FcεRI (kindly provided by Prof. Jean-Pierre Kinet)[8], were grown in the same conditions as above with additional G418 (Gibco/Thermo Fisher Scientific) antibiotic selection.

Human tissue immunohistochemistry

Normal tissue and melanoma patient tissue microarrays (US Biomax) and paraffin-embedded human tissue sections were stained with either a mouse monoclonal anti-CSPG4 IgG antibody (clone LHM02, Abcam; as previously described[9]) followed by biotinylated goat anti-mouse IgG (Vector labs, BA-9200-1.5), or with biotinylated anti-CSPG4 IgE (clone 225.28[10,11]). Anti-CSPG4 antibodies were detected by VECTOR Red Alkaline Phosphatase Substrate Kit using Levamisole (Vector Labs). Sections were counterstained with Mayer's hematoxylin solution (Sigma Aldrich), mounted in DPX mounting solution and visualized on a Zeiss Axiophot microscope using 10x and 20x magnification lenses (Carl Zeiss) and NIS-Elements imaging software (Nikon) or a tissue image analyzer (Hamamatsu). Absent, low, intermediate, and high CSPG4 expression was defined as 0%, <25%, 25-75% and >75% positively stained cells, respectively. Samples described as CSPG4 positive are comprised of low, intermediate and high-expressing tissues.

CSPG4 IgE specificity and cross-reactivity

Human CSPG4 knock-out (KO) cells: Human CSPG4 knock-out cell lines were generated using CRISPR-Cas9 technology on A375 and A2058 cells. In brief, phosphorylated and annealed CSPG4-targeting sgRNA (AAGTCTGGCCAACATAGTCA) was cloned into target vector px459-pSpCas9(BB)-2A-Puro (Addgene #62988) as previously described.[12] Cells were transfected using Lipofectamine, selected with Puromycin and single-cell cloned by serial dilution. Binding of our CSPG4 IgE (clone 225.28), and a commercial anti-CSPG4 IgG (REA989) to these cells was evaluated via flow cytometry.

Mouse CSPG4 expressing cells: Mouse CSPG4 expression by tumor-derived mouse melanoma cell lines obtained from the tamoxifen-inducible TyrCreER(t2)/Rac1(P29S)-LSL/BRaf(V600E)-Loxp/Trp53fl mouse model (JDGX strain)[13] was confirmed using a commercial anti-mouse

CSPG4 IgG (REA989) by flow cytometry and Western Blot. In parallel, cross-reactivity of our CSPG4 IgE (clone 225.28) was evaluated by flow cytometry.

Control IgE Antibodies

Her2 IgE which shares the same variable domains as Trastuzumab on an IgE domain backbone,[14] the folate receptor alpha-specific FRα IgE (MOv18 IgE)[15], and 4-hydroxy-3-nitro-phenacetyl (NIP) IgE specific for the hapten NIP [16] were prepared as previously described.

***In vitro* and *ex vivo* assays**

Direct effects of CSPG4 IgE: Adhesion of cancer cells to fibronectin-coated plates (Sigma) after 30 minutes;[17] cancer cell migration through a transwell system after 36 hours, and cancer cell invasion through a collagen layer after 48 hours (both using QCM ECMatrix Cell Invasion Assay kit (Merck)) were measured.

RBL-SX38 cell degranulation assay: β-hexosaminidase release was measured in culture supernatants of RBL-SX38 rat basophilic leukemia mast cells expressing human αβγ2 tetrameric FcεRI. Cells were seeded onto 96-well round bottom plates (Nunc, Roskilde, Denmark) at a concentration of 1 x 10^4 cells/well in 100µl media and incubated at 37°C/5% CO_2. The following controls were used: unstimulated cells; Triton X-100 lysed cells; hapten specific NIP IgE (AbD Serotec) with or without polyclonal rabbit anti-IgE (Dako). To investigate whether IgE-mediated interaction between mast cells and cancer cells could trigger mast cell degranulation, cells were sensitized with CSPG4 IgE, NIP IgE (200 ng/ml) or PBS and incubated at 37°C for 1 hour, washed 3 times in HBSS, 1% BSA (Invitrogen) stimulation buffer and stimulated for 30 minutes at 37°C with 100µl of 3x10^4 tumor cells per well. For evaluation of the potential for type I hypersensitivity, IgE-sensitized RBL-SX38 cells were incubated for 30 minutes at 37°C with 100µl human sera from healthy volunteers or melanoma patients. All conditions were tested in triplicate. 50µl cell culture supernatant (diluted 1:1 in stimulation buffer) and transferred to wells of 96-well plates with 50µl fluorogenic substrate (1mM 4-methylumbelliferyl N-acetyl-β-D-glucosaminide 0.1% DMSO, 0.1% Triton X-100, 200mM citrate pH 4.5) and incubated for 2 hours in the dark at 37°C. Reactions were quenched with

0.5M Tris and read on Fluostar Omega microplate reader (BMG Labtech) (350nm excitation, 450nm emission).

Three-color flow cytometric ADCC/ADCP tumor cell killing assay: Tumor cells were pre-labelled one day prior to assay with Carboxyfluorescein Succinimidyl Ester (CFSE) (Molecular Probes (Life Technologies)). Briefly, tumor cells were detached with 0.5M EDTA for up to 10 minutes, washed by centrifugation (1200rpm for 5 minutes) in standard media and then in serum-free Hank's balanced salt solution (HBSS, Life Technologies). Per $1x10^6$ tumor cells, 0.75µl of 0.5µM CFSE was incubated for 10 minutes at 37°C. Cells were washed with RPMI 1640 + GLUTAMAX, then centrifuged as above for 5 minutes. The cell pellet was resuspended in RMPI 1640 + GLUTAMAX media and returned to culture overnight. The following day, CFSE labelled cells were detached, washed, counted and resuspended to $1x10^6$ cells/ml. Control samples were either given no antibody or incubated with non-specific isotype control IgE. Test samples were incubated with 5 µg/ml of CSPG4 IgE. Following a wash with FACS buffer (PBS supplemented with 5% BSA), 100µl human effector cells and 100µl tumor cells (Effector:Target ratio of 3:1 for U937 and primary monocytes, 20:1 for PBMCs or monocyte-depleted PBMCs) were added to each tube with/without antibodies and mixed cell populations were incubated for 3 hours at 37°C/5% CO_2. After incubation, cells were washed and incubated for 20 minutes at 4°C with 2 µg/ml APC-conjugated anti-CD89 antibody (BD Bioscience) to label immune cells. Following a further wash, dead cells were labelled for 2 minutes at 4°C with DAPI (1:10000, Life Technologies) to label dead cells. Cells were washed and resuspended in FACS buffer and samples were acquired on a flow cytometer (FACSCanto II, Becton Dickinson). Representative flow cytometer plots and gating strategy for assessing tumor cell death are shown in Supplementary Figure 5a. Gate R1 is the total $CFSE^+$ tumor cell population. Gate R2 is $CFSE^+/APC^+$ cells, which indicate phagocytosis of $CFSE^+$ tumor cells by APC^+ effector cells. Gate R3 is $CFSE^+/DAPI^+$ dead tumor cells. The percentage of ADCC and ADCP is calculated using the cell counts in these regions, and with the following equation: ADCC % = $[(x + R3)/(R1\ control)] \times 100$; ADCP % = $[(R2 / (R1\ control)) \times 100$ [Key: R1 control = mean number of $CFSE^+$ tumor cells from the control tubes; R1 = number of $CFSE^+$ tumor cells; R2 = number of APC^+ tumor cells; R3 = number of $DAPI^+$ tumor cells; x = R1 control - R1].

ADCC in the presence of a kinase inhibitor: Prior to co-culture of immune effector cells and target cells, primary monocytes were incubated with PF-431396 kinase inhibitor (Sigma) for 20 minutes. Subsequently, monocytes were washed in media and then incubated with effector cells to detect ADCC/ADCP as above.

Real-time PCR of monocytic cells stimulated with IgE: Human monocytic U937 cells were pre-stimulated for 48 hours with 50 ng/ml of IL-4, in order to upregulate CD23 cell surface expression. Cells were seeded $1.5x10^5$ cells per well in 1ml complete RPMI and incubated with 5 µg/ml control NIP IgE or CSPG4 IgE for 30 minutes, at 37°C. Following centrifugation, supernatants were removed, and IgE-primed cells were resuspended in 1ml of 5 µg/ml polyclonal anti-IgE antibody and incubated for 1 hour at 37°C. Following centrifugation, supernatants were removed, cells were resuspended in lysis buffer (RLT+ß-ME) and stored at-80°C. Real-time PCR (qPCR) was performed using Taqman gene expression mastermix and the following probes: GAPDH-VIC housekeeping gene control (Hs99999905_m1; Thermo Fisher Scientific) and TNFa-FAM (Hs0113624 g1; Thermo Fisher Scientific). PCR cycles: 45 cycles of 2 minutes at 50°C, 10 minutes at 95°C, 15 seconds at 95°C and 1 minute at 60°C were run on the 7900HT Fast Real-time PCR system (Thermo Fisher Scientific) and data analysis performed with RQ Manager 1.2 software (Applied Biosystems).

Primary monocyte isolation and stimulation: Primary monocytes were isolated from healthy volunteer PBMCs by negative selection (Pan Monocyte Isolation Kit, Miltenyi, or RosetteSep Human Monocyte Enrichment Cocktail, STEMCELL Technologies). Monocytes were incubated at 1×10^6 cells/mL with 5 µg/mL IgE, or complete RPMI, for 1 hour at 37°C, 5% CO_2. Following extensive washing, FcεRI cross-linking was induced with 10 µg/mL polyclonal goat anti-human IgE (Abcam) at 37°C. Cells were washed after 1 hour cross-linking at 37°C, 5% CO_2, re-suspended in complete RPMI and incubated for 24 h at 37°C, 5% CO_2. Cytokine release in cell culture supernatants was analyzed by ELISA. Cell surface marker expression of stimulated monocytes was evaluated by flow cytometry using Fc receptor Block (BD Biosciences), viability DAPI stain (BioLegend), anti-CD14-BUV395 (clone: MøP9); BD Biosciences), anti-CD40-BV421 (clone: 5C3; BioLegend), anti-CD80-PE (clone: 2D10; BioLegend), anti-CD86-BUV737 (clone: 2331 (FUN-1); BD Biosciences), anti-CD163-APC (clone: GHI/61; BioLegend), anti-CCR2-BV605 (clone: K036C2; BioLegend), anti-PDL1-FITC (clone: MIH3; BioLegend) and

anti-HLA-DR-Alexa Fluor 700 (clone: L243, BioLegend). Cells were incubated with Fc block for 10 min at room temperature, washed and incubated again with the appropriate antibodies. After 30 min incubation at 4°C, the cells were washed, and 107 viable monocytes were acquired.

Primary monocyte A2058 co-cultures in the presence of CSPG4 IgE: Primary monocytes were plated at a 3:1 ratio with A2058 cells in the presence of CSPG4 IgE, or isotype control IgE. Supernatants were collected and secreted cytokines/chemokines (IL-1β, IL-4, IL-6, IL-10, IL-23, TNF (Mabtech) and CCL-2/MCP-1 (Invitrogen)) were measured using cytokine ELISA kits following the manufacturer's instructions. Plates were read using a Flurostar Omega Spectrophotometer (BMG Labtech).

Monocyte depletion, and monocyte-depleted PBMC characterization: Monocytes were depleted from whole unfractionated human blood using RosetteSep Human Monocyte Depletion Cocktail (STEMCELL Technologies). Cell surface marker expression of complete PBMCs and monocyte-depleted PBMCs were evaluated by flow cytometry using Fc receptor Block (BD Biosciences), Near-IR viability stain (ThermoFisher), anti-CD14-BUV395 (clone: MøP9; BD Biosciences), anti-CD45-FITC (clone: HI30; BioLegend), and anti-CD89-APC (clone: A59; BD Bioscience). Cells were incubated with Fc block for 10 min at room temperature, washed and incubated again with the appropriate antibodies. After 30 min incubation at 4°C, the cells were washed, and acquired.

Basophil activation test (BAT): Activation of basophils was analyzed by measurement of CD63 expression using the Flow2 CAST kit (BÜHLMANN Laboratories AG), in accordance with the manufacturer's instructions. 100µl unfractionated whole blood were incubated with 50µl stimulation buffer (Bühlmann Laboratories AG) and 100µl of one of the following stimuli: anti-FcεRI (Bühlmann Laboratories AG), anti-IgE antibody (Agilent Dako), fMLP (Bühlmann Laboratories AG), CSPG4 IgE, or control IgE (at 3.5 µg/ml final concentration, prepared in house). In the case of hapten-specific anti-NIP IgE, cross-linking with NIP-BSA (at 20 µg/ml, 5 NIP to BSA ratio, in-house) was included. In the case of CSPG4-expressing cells (WM1366, WM115, WM1361), 50,000 cells in 100µl stimulation buffer were included at an estimated ratio of 50:1 CSPG4-expressing cells to basophils in unfractionated whole blood (based on 10,000 basophils/ml blood). Lastly, all samples were stained with 20µl anti-CCR3-PE and anti-

CD63-FITC staining cocktail (Bühlmann Laboratories AG) and incubated at 37°C for 30 minutes (unless otherwise stated up to 8 hours) in a 5% CO_2 incubator. Following a further incubation with 2ml red blood cell lysis (Bühlmann Laboratories AG) for 10 minutes at room temperature, samples were centrifuged at 500G for 5 minutes, and cell pellets were resuspended in 150µl acquisition buffer (Bühlmann Laboratories AG). Samples were acquired on a flow cytometer (FACSCanto II, Becton Dickinson) whereby the CCR3-PE^{high}/SSC^{low} basophil population was identified and analyzed for CD63 expression as a marker of activation.

In vivo models of melanoma

Subcutaneous A375 human xenograft model with IgE and healthy volunteer peripheral blood immune cells: NSG mice were subcutaneously injected with 5×10^5 A375 melanoma cells in 150µl PBS. At day 5, 10×10^6 PBLs and 10mg/kg antibody were injected intravenously. Antibody treatment was injected three more times (day 12, 18 and 25), or once more (day 13) at the same 10mg/kg dose in 150µl PBS. Control groups were treated with 10×10^6 PBLs on day 5 and 150µl PBS on treatment days. Subcutaneous tumor growth was monitored and measured with calipers. Tumor size (mm^3) calculated using the following formula: $mm^3 = d^2 \times (D/2)$ (d = smallest diameter of tumor; D = largest diameter of tumor).

Development of A375 lung tumor model treated with IgE and healthy volunteer peripheral blood immune cells: NSG mice injected intravenously with 5×10^6 healthy volunteer PBMCs and 5×10^5 A375 cells pre-incubated with 500µg of respective antibody. Antibody treatment injected intravenously at day 1 and day 2 at 10mg/kg doses. At day 28 animals were sacrificed and lungs were stained with a solution of 15% v/v India ink, washed in Milli-Q water, and destained in Fekete solution to visualize lesions. The number of tumors per lung lobe and the proportional occupancy of tumors in the lung area were measured using a Nikon SMZI500 stereo microscope and NIS Elements Basic Research software (Nikon UK Ltd.).

Subcutaneous A375 tumors treated with IgE and patient-derived peripheral blood immune cells: NSG mice were subcutaneously injected with 5×10^5 A375 melanoma cells in 150µl PBS. At day 5, 10×10^6 melanoma patient-derived PBMCs and 10mg/kg antibody were injected intravenously. Antibody treatment was injected three more times (day 12, 18 and 25) at the

same 10mg/kg dose in 150µl PBS. Control groups were treated with $10x10^6$ PBMCs on day 5 and 150µl PBS on treatment days. Tumor growth was monitored and measured as above.

PDX with autologous patient PBLs: NSG mice were transplanted with equal sizes of patient-derived melanoma tumors, obtained from two donors (stage III and IV) by surgical procedure. Mice were anaesthetized and the surgical area was cleared of hair and disinfected with Povidon-Iodide solution (Betaisodona Mundipharm). A 1cm incision was made above the abdomen. Tumor was placed between the dermis and peritoneum, whilst avoiding perforating the peritoneum. Incision was closed using Vetbond tissue adhesive (3M) and bandaged. At the same time, mice were injected intravenously with $10x10^6$ human PBLs from the same donor (autologous) and 10mg/kg of antibody (or control saline). Subsequent injections of antibody were given every 7 days. Tumor growth was monitored and measured as above.

Immunohistochemical and immunofluorescence analyses of human xenograft samples

Retention of CSPG4 expression *in vivo*: Following *in vivo* tumor growth, retention of CSPG4 expression was evaluated in excised tumors by immunofluorescence using CSPG4 IgE, or NIP IgE as an isotype control, followed by goat anti-human IgE-FITC secondary antibody (Jackson ImmunoResearch), and by immunohistochemistry using biotinylated CSPG4 IgE and detected using alkaline phosphatase (AP) substrate buffer. Sections were counterstained by Mayer's hematoxylin solution (Sigma Aldrich) Human immune cell engraftment in mouse spleens was confirmed by flow cytometry using mouse anti-human CD45 antibody (eBioscience).

Tumor immune infiltration: Immune cell infiltration into excised tumors following CSPG4 IgE or control IgE treatment was studied using mouse anti-human CD45 (eBioscience) and mouse anti-human CD68 (DAKO) antibodies detected with biotinylated goat anti-mouse IgG (Vector labs) with horseradish peroxidase-conjugated IgG Fc-specific antibody (DAKO) and visualized with DAB chromogenic substrate (DAKO), following the manufacturer's instructions. This was followed by Mayer's hematoxylin solution counterstaining (Sigma Aldrich).

Transcriptomic analysis of excised A375 tumors

Human Ref-6 and Ref-12 BeadChips (Illumina, Ambion) were used to generate RNA expression data from excised A375 tumors (n=5 PBS control; n=4 CSPG4 IgE treatment). Each array contains over 50000 probe sets representing approximately 40000 human genes.

Differential gene expression in tumors from treatment and control animals were first studied for monocyte and macrophage gene signatures. Signature markers were genes, with FDR <= 0.05, derived from a single-cell RNA-seq dataset from treatment naive patients (GSE123139), and clustered using Seurat package and function *FindAllMarkers()* (parameters: only.pos = TRUE, min.pct = 0.25, logfc.threshold = 0.25). Clusters represent the cell identity as described by Li et al..[18]

Additional, differentially expressed genes between treatment and control were identified using the package limma (3.48.3).[19] The list of all genes, ranked according to fold change, was used to calculate enrichment of gene sets within Reactome (7.4)[20] using package fgsea (1.18.0)[21] with random seed set to 42. 25 gene sets of interest were visualized using ggplot2 (3.3.5).[22] R version 4.1.1 was used.

In vivo **antibody clearance in subcutaneous A375 model**

Clearance of CSPG4 IgE and IgG antibodies administered at 10mg/kg was monitored in mouse serum for up to 50 hours post injection using human IgE or IgG ELISAs (developed in house). Briefly, Maxisorp plates (Nunc) were coated with polyclonal anti-human IgE (DAKO) or anti-human IgG (AbD Serotec) antibodies, respectively, each diluted 1:1000 in carbonate-bicarbonate buffer. Plates were blocked with 2% milk-PBS solution. Standard curves (human IgE, WHO and human IgG, Sigma, respectively) and mouse serum samples were incubated on the plates overnight (for IgE) or for 2 hours (for IgG). Following washing with PBS-Tween solution, bound antibodies were detected with anti-human IgE-peroxidase antibody (Sigma), or anti-human IgG-HRP antibody (Jackson ImmunoResearch), respectively, followed by development with OPD and measurement at 492nm.

References:

1 Sievers, F. *et al.* Fast, scalable generation of high-quality protein multiple sequence alignments using Clustal Omega. *Mol Syst Biol* **7**, 539, doi:10.1038/msb.2011.75 (2011).

2 Goujon, M. *et al.* A new bioinformatics analysis tools framework at EMBL-EBI. *Nucleic Acids Res* **38**, W695-699, doi:10.1093/nar/gkq313 (2010).

3 *The Human Protein Atlas. CSPG4, Cancer CSPG4 expression*, <https://www.proteinatlas.org/ENSG00000173546-CSPG4/pathology> (

4 Uhlen, M. *et al.* A pathology atlas of the human cancer transcriptome. *Science* **357**, doi:10.1126/science.aan2507 (2017).

5 Tang, Z. *et al.* GEPIA: a web server for cancer and normal gene expression profiling and interactive analyses. *Nucleic Acids Res* **45**, W98-W102, doi:10.1093/nar/gkx247 (2017).

6 Goldman, M. J. *et al.* Visualizing and interpreting cancer genomics data via the Xena platform. *Nat Biotechnol* **38**, 675-678, doi:10.1038/s41587-020-0546-8 (2020).

7 Sundstrom, C. & Nilsson, K. Establishment and characterization of a human histiocytic lymphoma cell line (U-937). *Int J Cancer* **17**, 565-577, doi:10.1002/ijc.2910170504 (1976).

8 Wiegand, T. W. *et al.* High-affinity oligonucleotide ligands to human IgE inhibit binding to Fc epsilon receptor I. *J Immunol* **157**, 221-230 (1996).

9 Williams, I. P. *et al.* In vivo safety profile of a CSPG4-directed IgE antibody in an immunocompetent rat model. *MAbs* **12**, 1685349, doi:10.1080/19420862.2019.1685349 (2020).

10 Neri, D. *et al.* Recombinant anti-human melanoma antibodies are versatile molecules. *J Invest Dermatol* **107**, 164-170, doi:10.1111/1523-1747.ep12329566 (1996).

11 Ghose, T. *et al.* Regression of human melanoma xenografts in nude mice injected with methotrexate linked to monoclonal antibody 225.28 to human high molecular weight-melanoma associated antigen. *Cancer Immunol Immunother* **34**, 90-96, doi:10.1007/BF01741341 (1991).

12 Ran, F. A. *et al.* Genome engineering using the CRISPR-Cas9 system. *Nat Protoc* **8**, 2281-2308, doi:10.1038/nprot.2013.143 (2013).

13 Lionarons, D. A. *et al.* RAC1(P29S) Induces a Mesenchymal Phenotypic Switch via Serum Response Factor to Promote Melanoma Development and Therapy Resistance. *Cancer Cell* **36**, 68-83 e69, doi:10.1016/j.ccell.2019.05.015 (2019).

14 Ilieva, K. M. *et al.* AllergoOncology: Expression platform development and functional profiling of an anti-HER2 IgE antibody. *Allergy* **74**, 1985-1989, doi:10.1111/all.13818 (2019).

15 Gould, H. J. *et al.* Comparison of IgE and IgG antibody-dependent cytotoxicity in vitro and in a SCID mouse xenograft model of ovarian carcinoma. *Eur J Immunol* **29**, 3527-3537, doi:10.1002/(SICI)1521-4141(199911)29:11<3527::AID-IMMU3527>3.0.CO;2-5 (1999).

16 Neuberger, M. S. *et al.* A hapten-specific chimaeric IgE antibody with human physiological effector function. *Nature* **314**, 268-270, doi:10.1038/314268a0 (1985).

17 Wang, X. *et al.* CSPG4 protein as a new target for the antibody-based immunotherapy of triple-negative breast cancer. *J Natl Cancer Inst* **102**, 1496-1512, doi:10.1093/jnci/djq343 (2010).

18 Li, H. *et al.* Dysfunctional CD8 T Cells Form a Proliferative, Dynamically Regulated Compartment within Human Melanoma. *Cell* **176**, 775-789 e718, doi:10.1016/j.cell.2018.11.043 (2019).

19 Ritchie, M. E. *et al.* limma powers differential expression analyses for RNA-sequencing and microarray studies. *Nucleic Acids Res* **43**, e47, doi:10.1093/nar/gkv007 (2015).

20 Subramanian, A. *et al.* Gene set enrichment analysis: a knowledge-based approach for interpreting genome-wide expression profiles. *Proc Natl Acad Sci U S A* **102**, 15545-15550, doi:10.1073/pnas.0506580102 (2005).

21 Korotkevich, G. *et al.* Fast gene set enrichment analysis. *bioRxiv*, 060012, doi:10.1101/060012 (2021).

22 Wickham, H. *Ggplot2 : elegant graphics for data analysis*. 2 edn, Vol. 1 189-201 (Springer Cham, 2016).
